# Network evaluation from the consistency of the graph structure with the measured data

**DOI:** 10.1186/1752-0509-2-84

**Published:** 2008-10-01

**Authors:** Shigeru Saito, Sachiyo Aburatani, Katsuhisa Horimoto

**Affiliations:** 1 Biological Network Team, Computational Biology Research Center (CBRC), National Institute of Advanced Industrial Science and Technology (AIST), 2-42 Aomi, Koto-ku, Tokyo 135-0064, Japan; 2 Chem & Bio Informatics Department, INFOCOM CORPORATION, Mitsui Sumitomo Insurance Surugadai Annex Building, 3-11, Kanda-surugadai, Chiyoda-ku, Tokyo 101-0062, Japan

## Abstract

**Background:**

A knowledge-based network, which is constructed by extracting as many relationships identified by experimental studies as possible and then superimposing them, is one of the promising approaches to investigate the associations between biological molecules. However, the molecular relationships change dynamically, depending on the conditions in a living cell, which suggests implicitly that all of the relationships in the knowledge-based network do not always exist. Here, we propose a novel method to estimate the consistency of a given network with the measured data: i) the network is quantified into a log-likelihood from the measured data, based on the Gaussian network, and ii) the probability of the likelihood corresponding to the measured data, named the graph consistency probability (*GCP*), is estimated based on the generalized extreme value distribution.

**Results:**

The plausibility and the performance of the present procedure are illustrated by various graphs with simulated data, and with two types of actual gene regulatory networks in *Escherichia coli*: the SOS DNA repair system with the corresponding data measured by fluorescence, and a set of 29 networks with data measured under anaerobic conditions by microarray. In the simulation study, the procedure for estimating *GCP *is illustrated by a simple network, and the robustness of the method is scrutinized in terms of various aspects: dimensions of sampling data, parameters in the simulation study, magnitudes of data noise, and variations of network structures.

In the actual networks, the former example revealed that our method operates well for an actual network with a size similar to those of the simulated networks, and the latter example illustrated that our method can select the activated network candidates consistent with the actual data measured under specific conditions, among the many network candidates.

**Conclusion:**

The present method shows the possibility of bridging between the static network from the literature and the corresponding measurements, and thus will shed light on the network structure variations in terms of the changes in molecular interaction mechanisms that occur in response to the environment in a living cell.

## Background

The knowledge-based approach to construct biological network models is recognized as one of the most promising advances in computational biology [1]. In this approach, the causal relations between biological molecules are described as a directed graph, based on the interaction information extracted from a large number of previous reports, in a manual or automatic manner [2,3]. Since each relation has been identified by experimental studies, the existence of edges in the network model is supported by strong evidence. Due to the high reliability of each relation, many network models, even those with large, complex structures, have been constructed for various biological phenomena by a knowledge-based approach [4-6]. Note that a network generated by a knowledge-based approach is a mixture of molecular relationships identified by experimental studies under different conditions. Indeed, it is well known that the relationships between the molecules in a living cell change dynamically, depending on the cellular environment. Fortunately, an abundance of such information about molecular interactions under different conditions has been obtained by measuring them on a genomic scale, due to recent advances in experimental techniques, and the information about the interactions is available at various web sites [7]. Thus, we can evaluate the consistency of the knowledge-based network structure by the available information about the data measured under the different conditions. Although the inference of static network structures from the data has been intensively studied by various approaches, such as the Bayesian network [8], the dynamic Bayesian network [9], the Boolean network [10], and the graphical Gaussian model [11], the consistency evaluation will be useful to trace the dynamic network structure variations reflecting the molecular relationships that change coordinately in response to the cellular environment.

The consistency evaluation between the network structure and the measured data is well known in statistics as the test for causal hypotheses by using the measured data. The origin of the test for causal hypotheses is attributed to path analysis [12]. Unfortunately, the importance of this cornerstone research was not recognized for a long time, but the natural extension of path analysis has been established as the well-known structural equation model (SEM) [13]. Indeed, the SEM has been utilized recently in various fields, in accordance with increased computer performance. However, the SEM without any latent variables, which is a natural assumption for its application to biological networks, sometimes has difficulties in the numerical calculation of the maximum likelihood for the observed data. To overcome the problem with this calculation, the d-sep test [14] has been developed, based on the concept of d-separation in a directed acyclic graph (DAG) [15]. Note that the graph consistency with the data in the d-sep test is considered by focusing on the absence of edges in the graph [16,17].

Recently, linear regression was applied to reconcile the gene regulatory network with the corresponding data [18]. This application is based on the concept that the entire network of gene regulation can be divided into a few network motifs, with a two-layer relationship between the transcription factors and their regulated genes [19]. Indeed, the division of the entire network into a small and simple network enables us to utilize the standard statistical tests in linear regression for the consistency of the gene relationships with the measured data. Unfortunately, the linear regression is limited to the two-layer relationships, and subsequently, its application is constrained to the simple structures of gene regulatory networks.

In this study, we propose a new method for estimating the consistency of a causal graph with the measured data, in combination with the Gaussian network (GN) [20] and the generalized extreme value distribution (GEV) [21]. The present study partly exploits the previous study [18] about the consistency between the network motif with two-layer gene relationships and the measured data. However, instead of the network motifs with simple structures, here we consider rationally complicated network structures based on the graphical model, and its consistency with the data is expressed as a probability, referred to as the graph consistency probability (*GCP*). The performance of the present method is examined by artificial networks with various structures and actual data measured in *Escherichia coli*. Furthermore, the merits and pitfalls of our method are discussed in terms of its possible utility with various actual issues and methodologies, in comparison with previous methods.

## Results and discussion

### Calculation of Graph Consistency Probability (*GCP*)

**Figure 1:**
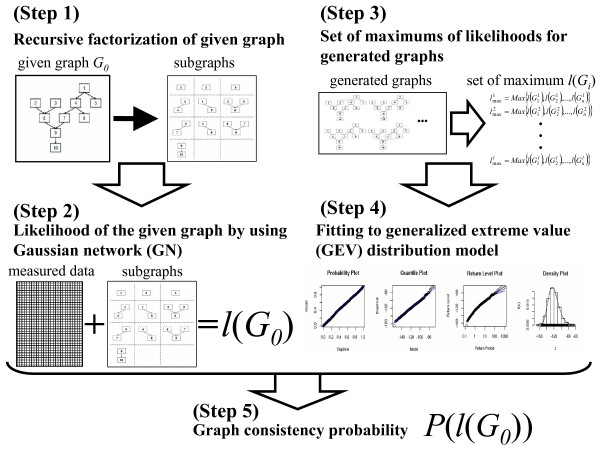
**Flow of the calculation of graph consistency probability**. The calculation is composed of five steps (see details in the text).

We will illustrate the procedure for calculating the graph consistency probability (*GCP*) with a simple graph, *G*_0_, which is a directed acyclic graph with ten nodes and nine edges, and with the corresponding data that are artificially generated on the assumption that the data noise follows the normal distribution. The procedure for calculating the *GCP *is composed of five steps, as schematically shown in Fig. 1 (see details of the mathematical description in the Materials and Methods and the additional file 1: Details of the schematic description of the procedure).

At the first step, the given graph, *G*_0_, is recursively factorized into the subgraphs, according to the parent-descent relationships in DAG [15]. By recursive factorization, *G*_0 _is rationally divided into 10 subgraphs, based on the parent-descent relationships in the directed graph.

At the second step, we calculated the log-likelihood of the given graph, *l*(*G*_0_), with the corresponding data by a Gaussian network model [20]. To calculate *l*(*G*_0_), here, we generated the data {X_*kl*_} for each node with 50 sample dimensions, i.e., for *k *= 1,2,...,10 and *l *= 1,2,...,50, instead of the actual data, by the structural equations (see details in Methods). The *l*(*G*_0_) of the given graph was then calculated to -31.14. We will estimate the probability of *l*(*G*_0_), *GCP*, by the following three steps.

At the third step, we generated the graphs based on the given graph, and then calculated the log-likelihoods of the generated graphs according to the two preceding steps.

(1) We generated 50 random graph sets, {*G*_*i*_}, to form a data set, in which each graph has the same number of nodes and edges, but with different connections from those of *G*_0_.

(2) 50 corresponding log-likelihoods of {*G*_*i*_} were calculated according to the first and second steps. Among the 50 log-likelihoods, the maximum of the log-likelihood, *l*(*G*_*max*_), is selected.

(3) The above procedure is iterated 1000 times to finally obtain 1000 values of *l*(*G*_*max*_). In this step, the dimensions of the sampling data, the number of graphs in one set, and that of the iterations to select *l*(*G*_*max*_) are changeable parameters, and the robustness of our method with them will be evaluated in the following sections.

At the fourth step, we fit the log-likelihoods calculated in the third step to the GEV model. The maximization of the GEV log-likelihood leads to the following estimate:

$$\left(\hat{\mu },\hat{\sigma },\hat{\xi }\right)=(-126.58,13.15,-0.148),$$

for which the GEV log-likelihood is 4063.59. Although the maximum likelihood estimate for $\hat{\xi }$ is negative, corresponding to a bounded distribution, the *ξ *value larger than -0.5 indicates that the maximum likelihood functioned well for the estimation [21,22]. Furthermore, the goodness of fit can be visually diagnosed, using the three diagnostic plots for assessing the accuracy of the GEV model, fitted to the 1000 log-likelihoods data by the three parameterizations. Neither the probability plot nor the quantile plot gave any cause to doubt the validity of the fitted model: each set of plotted points was nearly linear. The return-level curve asymptotes to a finite level as a consequence of the negative estimate $\hat{\xi }$, and also provides a satisfactory representation of the empirical estimates. In addition, the corresponding density estimate seems consistent with the histogram of the data. Consequently, the four diagnostic plots lend support to the fitted GEV model.

At the final step, we estimated the *GCP *of the log-likelihood of the given graph based on the fitted GEV distribution. According to the GEV distribution, the *GCP *corresponding to *l*(*G*_0_) (= -31.14) was calculated to be less than 10^-10^. As a result, it is natural that the examined given graph was highly consistent with the data generated according to the graph structure.

### Robustness of the Present Method

The high performance of the present method described in the preceding subsection depends on a few parameters. By using the same network structure as in Fig. 1, we tested the robustness of the present method in terms of the dimensions of the analyzed data, the two parameters in generating artificial graphs for GEV, and the degree of noise in the data. Furthermore, the robustness of the network structure variation is tested by using the typical network structure in biological interactions in the following four subsections.

### Robustness in Terms of the Dimensions of the Analyzed Data

We test the robustness of our method in terms of the number of data samples for one variable (data dimension) that is smaller than the data dimension ({X_*i*_} for *i *= 1,2,...,50) in Fig. 1. This is because the experimental conditions are frequently limited, due to the technical difficulty of performing experiments for different growth conditions. Thus, small data dimensions are expected in the actual data.

We performed the same estimation of *GCP *as that in Fig. 1, by using the data with 15 and 30 dimensions, and in both cases, the present method operated well. The GEV fit well to the data: the estimated $\hat{\xi }$ was larger than -0.5: the estimated $\hat{\xi }$ values are -0.1132 for the 15-dimension data and -0.1332 for the 30-dimension data. In addition, the four GEV-diagnostic plots for assessing the accuracy of the GEV model show the validity of the fitted model in each case (see additional file 2: Robustness in terms of data dimensions). By the estimated GEV distributions, the *GCP*s in the two cases were less than 10^-4 ^and 10^-8^, respectively. The probability for the 30-dimension data was smaller than that for the 15-dimension data. Considering that the probability was 10^-10 ^in the 50-dimension data in the preceding section, this indicates that the resolution degree about the consistency is higher with larger dimensions.

### Robustness in Terms of Parameters in Generating the GEV Model

The GCP depends on two parameters in the graph generation for GEV: the number of graphs for selecting the maximum of the likelihood in one set of the generated graphs, *l*, and the number of iterations for sampling the maximum values from each set of generated graphs, *n*. In Fig. 1, *l *and *n *were set to 50 and 1000, and a total of 50,000 graphs were generated for GEV. Here, we examined the fitness of the log-likelihoods to GEV based on the graph shown in Fig. 1, with nine pairs of *l *and *n*: *l *was set to 25, 50 and 100, and *n *was set to 100, 500, and 1000. The total numbers of graphs for GEV ranged from 2500 to 100000, and all of the examinations with the above parameter pairs are provided in an additional file (see additional file 3: Robustness in terms of the parameters). Here, we focused on the case when fewer graphs are generated than the number in the default case. This is because a small number of generated graphs in each set and iterations may tend to violate the distribution of GEV, due to some biases in the graph generation.

In the comparison of (*l*, *n*) = (50, 100) and (25, 1000) with (50, 1000) in Fig. 1, the log-likelihoods calculated in the two cases were fitted to the GEV model. Indeed, the two $\hat{\xi }$ values were larger than -0.5: the estimated $\hat{\xi }$ values were -0.1545 in (*l*, *n*) = (50, 100) and -0.1670 in (*l*, *n*) = (25, 1000), respectively. The two sets of diagnostic plots for assessing the accuracy also showed the validity of the fitted model in each case (see additional file 3). A closer inspection revealed that the $\hat{\xi }$ value in the (50, 100) case was slightly less than that in the (25, 1000) case. This indicated that the number of graphs in each set, *l*, is more sensitive to the goodness of fitness than the number of iterations, *n*, regardless of the total number of generated graphs. At any rate, the present method operates well, even in the case of a relatively small number of generated graphs.

In general, the optimized values of *l *and *n *depend on the size of the examined graph, and may be expressed as the fraction to the total number of possible graphs with the same numbers of nodes and edges as those of the examined graph. Although the number of possible graphs composed of arbitrary numbers of labeled nodes and edges can be estimated asymptotically under some constraints on the edge connectivity [23], unfortunately, the total number of possible graphs, in which all of the nodes are connected to form one graph, is not still obtained. In the present stage, we should heuristically define *l *and *n *by diagnosing the goodness of fit to the GEV model.

### Robustness in Terms of the Magnitude of Noise in the Analyzed Data

We estimated the *GCP *in various noise ranges. For this purpose, the value of the standard deviation in the structural equations for data generation (*σ *= 0.1 in Fig. 1) was changed to three values (*σ *= 0.5, 1.0, and 2.0). By the same procedure as that in Fig. 1, we calculated 100 *GCPs *for the three ranges of standard deviations. Finally, the probabilities of the generated graphs were calculated.

**Figure 2:**
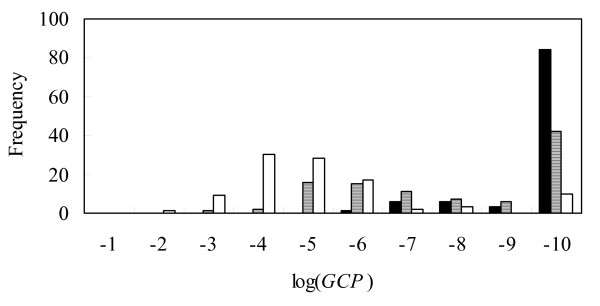
**Robustness in terms of the noise in measured data**. *GCP*(=*P*(*l*(*G*_0_))) for the graph in Fig. 1 was calculated with simulated data with distinct standard deviations, and the frequencies of *GCP*s are plotted against the probability degree. The horizontal axis indicates the log(*GCP*) value, and the vertical axis is its frequency: black-colored bar, *σ *= 0.5; gray-colored bar, *σ *= 1.0; and boxed bar, *σ *= 2.0.

The histograms of the *GCPs *in the three ranges of standard deviations are shown in Fig. 2. In this figure, 100 *GCPs *were plotted against the number of connections in the generated graphs that were different from those in the examined graph in the respective cases of standard deviations. In the cases of the two small standard deviations (*σ *= 0.5 and 1.0), less than 10^-10 ^of the *GCPs *emerged most frequently, but the most frequent *GCP *was found at 10^-4 ^in the case of the largest standard deviation (*σ *= 2.0). In the former two cases, the largest *GCP *was 10^-6 ^in *σ *= 0.5 and 10^-3 ^in *σ *= 1.0. Although some exceptional *GCPs *were also found, the present method operates well within the range of the two noise levels. In contrast, the last case shows the limitation of our method, in terms of the noise of the measured data. Careful preprocessing of the measured data may be required to apply our method to actual data. Note that the noise is amplified as the number of parents grows in the present simulation. For example, the standard deviation is (*α*_1_^2 ^+ *α*_2_^2 ^+ 1)*σ*, when a descent has two parents and *α*_*i *_is the path coefficient between the descent and the *i*-th parent. At any rate, the limitation of the present method in terms of the data noise can be examined by describing the histogram of the *GCP*, and was estimated between 1.0 and 2.0 for the graph in Fig. 1. In addition, we assumed that the distribution of the data noise also follows uniform and gamma distributions, and obtained similar results in terms of the robustness about the data noise (see additional file 4: Robustness in terms of the noise according to the gamma and uniform distributions).

### Robustness Regarding the Variation of the Network Structure

**Figure 3:**
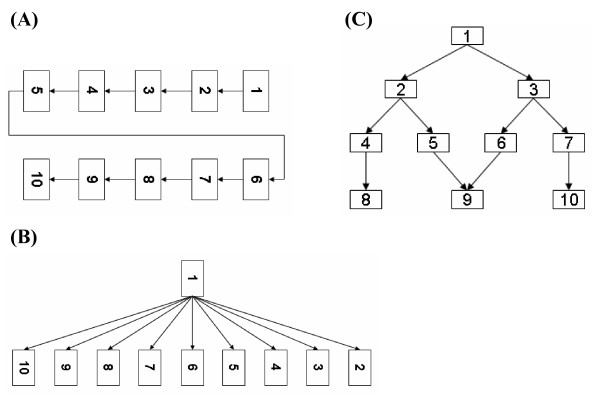
**Robustness regarding graph structure variation**. The calculation is composed of five steps (see details in the text). Three networks with typical structures in biology are examined in (A), (B), and (C). To generate the simulation data by structural equations, we set the standard deviation to 0.1 in all three graphs, and the path coefficients between the variables are as follows: (A) *α*_1,2 _= 0.6, *α*_2,3 _= 0.3, *α*_3,4 _= 0.1, *α*_4,5 _= 0.7, *α*_5,6 _= 0.8, *α*_6,7 _= 0.9, *α*_7,8 _= 0.2, *α*_8,9 _= 0.5, and *α*_9,10 _= 0.4; (B) *α*_1,2 _= 0.1, *α*_1,3 _= 0.2, *α*_1,4 _= 0.3, *α*_1,5 _= 0.4, *α*_1,6 _= 0.5, *α*_1,7 _= 0.6, *α*_1,8 _= 0.7, *α*_1,9 _= 0.8, and *α*_1,10 _= 0.9; and (C) *α*_1,2 _= 0.5, *α*_1,3 _= 0.7, *α*_2,4 _= 0.4, *α*_2,5 _= 0.8, *α*_3,6 _= 0.6, *α*_3,7 _= 0.3, *α*_4,8 _= 0.2, *α*_5,9 _= 0.1, *α*_6,9 _= 1.0, and *α*_7,10 _= 0.9. The value of log-likelihood and the parameters of GEV distribution in the respective networks are as follows: (A) *l*(*G*_0_) = 163.4805, *μ *= 89.8375, *σ *= 12.9694, and *ξ *= -0.1743; (B) *l*(*G*_0_) = 61.6096, *μ *= 3.0217, *σ *= 12.5220, and *ξ *= -0.1314; and (C) *l*(*G*_0_) = 124.8894, *μ *= 46.9002, *σ *= 12.1395, and *ξ *= -0.1406. See also the corresponding GEV plots at additional file 5: Robustness regarding the network structure variation.

We applied the present method to the three network structures shown in Fig. 3. The three networks are analogous to the typical structures of biological networks; the first is analogous to part of a chain reaction in a metabolic pathway, the second represents the simple structure of a gene regulatory network, and the third depicts a cascade in a signal transduction pathway. According to the connectivity in the network, the data were generated with the corresponding structural equations, and the present method was applied to estimate the graph consistency with the generated data.

The present method operated well in all of the network structures. Indeed, the log-likelihoods in the three networks fit well to the GEV (see statistics in the legend of Fig. 3, and additional file 5: Robustness regarding the network structure variation). In addition, the *GCP*s were very small: The *GCP*s of the three networks were less than 10^-11^, 10^-4^, and 10^-7^, respectively. Interestingly, the magnitudes of the *GCP*s may be related to the network structures. The *GCP *in Fig. 3B is relatively larger than the *GCP*s in Figs. 3A and 3C. This is because the present path coefficients between the 10 nodes were set at different values, but in the same order of digits. This indicates that the most similar data for respective variables were generated in Fig. 3B, and caused pseudo-correlations between the variables with no edges in the network in Fig. 3B. Although the performance for estimating the graph consistency may slightly decrease, depending on the number of two -layer relationships in the examined data, this simulation shows that the present method can be applied to various structures of networks.

### Examinations of Actual Graphs

We examined the performance of the present method with two sets of actual networks in *Escherichia coli *and the corresponding actual measured data. One set is a regulatory network for the SOS response system with the expression degrees of the constituent genes measured by fluorescence [24], and the other is 29 networks classified by gene functions, with the expression degrees under anaerobic conditions measured by microarray [25]. The former examination is a verification of the present method for an actual network with a size similar to the networks shown in Fig. 1, and the latter is a demonstration of a high-throughput search of network candidates, consistent with the data measured under particular conditions.

### Verification for a Simple Network

**Figure 4:**
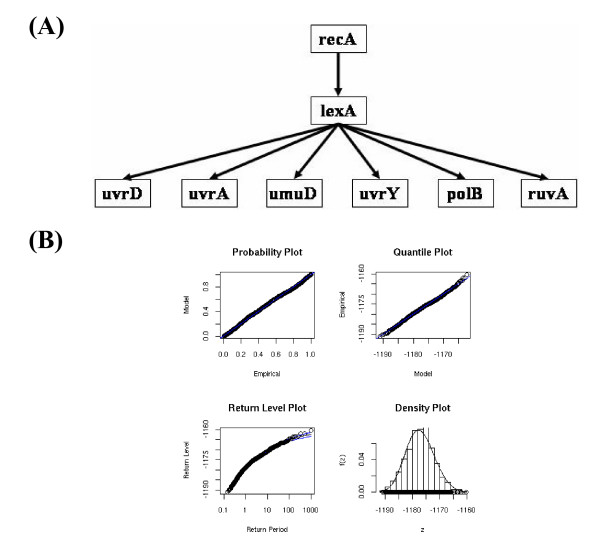
**Evaluation of the transcriptional network of the SOS DNA repair system in *Escherichia coli***. The network is schematically shown in (A), and the corresponding GEV plots and the box-plot are also shown in (B). The value of log-likelihood between the examined network and the measured data is -1168.453, and the parameters of GEV distribution are as follows: *μ*, -1179.079, *σ*, 4.957; *ξ*, -0.236. The data for the promoter activities of eight genes in the SOS system are cited from [24].

The gene network in the SOS system is schematically shown in Fig. 4A. The SOS DNA repair system in *Escherichia coli *is a well-characterized transcriptional network [26,27]. One of the SOS proteins, *RecA*, acts as a sensor of DNA damage, and a master repressor (*LexA*) binds sites in the promoter regions of these operons. The corresponding data to the constituent molecules in the network are the transcriptional activity of genes measured with real-time monitoring by means of low-copy reporter plasmids, in which a promoter controls green fluorescent protein [24].

The GEV plots with the likelihood values and the statistics are shown in Fig. 4B. The value of $\hat{\xi }$ was larger than -0.5, and the GEV plots were quite similar to those in Fig. 1. Indeed, each set of plotted points was nearly linear, and the return-level curve asymptotes to a finite level. In addition, the corresponding density estimate seems consistent with the histogram of the data. Consequently, the goodness of fitness in the actually measured data lends support to the GEV model.

The *GCP *of the SOS network with the corresponding measured data was estimated as 0.049, and the network structure was estimated to be consistent with the data measured from the examined network. However, the *GCP *was large in comparison with the *GCP*s in the simulation studies in the preceding sections. This is partly because the cyclic relationship of *RecA *is neglected in the examined network, and partly because most of the relationships in the examined network are composed of 2-layer relationships, due to the production of similar degrees of expression data, as in the situation in Fig. 3B. At any rate, the performance of the present method was verified by a well-known network, with a size similar to that in the simulation, and with the corresponding data measured by an experimental study.

### Demonstration for an Actual Network Set

We further tested the performance of our method for selecting the networks consistent with the data measured under specific conditions from many network candidates. Here, we arranged 29 regulatory networks in *Escherichia coli *and the corresponding gene expression profiles measured under anaerobic conditions (for details about the examined network reconstruction and the profile data, see Methods).

**Figure 5:**
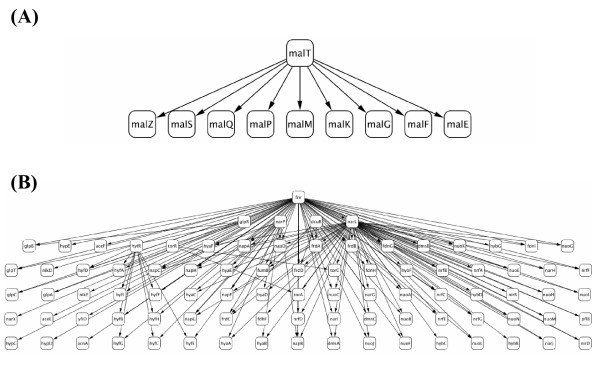
**Networks with 5% significance probability in graph consistency search**. By corresponding between the regulatory relationships and the gene functions in EcoCyc [44], 29 regulatory networks were reconstructed, and their consistency with the expression profiles measured under anaerobic conditions (accession number GSE1107 in NCBI Gene Expression Omnibus (GEO); ) [25] was examined. Among the 29 regulatory networks, two networks showed 5% significance probability: the network related with carbon compounds (EcoCyc ID: C9449_11) (A) and that with anaerobic respiration (EcoCyc ID: C9490_1) (B). The details of the network structures of the 29 regulatory networks are shown in the additional file 6: the 29 network structures analyzed in the present study.

Table 1 shows the analyzed networks and the corresponding graph consistency probabilities of the 29 networks (see additional file 6: the 29 network structures analyzed in the present study). When we set the significance probability to 5%, only two networks (Nos. 14 and 28) in Fig. 5, which are composed of regulatory gene pairs related to carbon compounds and anaerobic respiration, were selected among the 29 networks. As seen in the figure, the network structures are quite different. The network related to the carbon compounds in Fig. 5A shows a relatively simple structure that is a two-layer relationship between one TF and its nine regulated genes. In contrast, the other network related to anaerobic respiration in Fig. 5B has a highly complicated form, with 89 nodes and 161 edges. The selection of the two networks can be interpreted in terms of biological functions, as described below.

**Table 1:** Consistency of the twenty-nine networks with expression profiles measured under anaerobic conditions in *Escherichia coli*

No.	ID	Description	node	edge	*GCP*
1	C9333	detoxification	6	8	1.000
2	C9448	amino acids	6	9	1.000
3	C9449	carbon compounds	6	9	1.000
4	C9426	colanic acid (M antigen)	6(7)	9(11)	1.000
5	C9509	operon	6(7)	9(11)	1.000
6	C9448, C9462	amino acids, formyl-THF biosynthesi	7	10	1.000
7	C9449	carbon compounds	8(9)	7(8)	1.000
8	C9331	motility, chemotaxis, energytaxis	9	8	0.998
9	C9340	flagella	9	8	0.647
10	C9362	nucleoproteins, basic proteins	9	8	0.925
11	C9401	tryptophan	9	8	1.000
12	C9449	carbon compounds	9	8	1.000
13	C9376	cytoplasm	10	9	1.000
14	C9449	**carbon compounds**	10	9	**0.006**
15	C9449	carbon compounds	10	11	0.976
16	C9337	SOS response	11	10	0.127
17	C9354	DNA repair	11	10	0.068
18	C9383	arginine	11	10	1.000
19	C9474	nucleotide and nucleoside conversion	11	15	0.378
20	C9493	fermentation	11	10	1.000
21	C9376	cytoplasm	12	11	0.302
22	C9393	isoleucine/valine	13	12	1.000
23	C9420	purine biosynthesis	13	12	1.000
24	C9394	leucine	14	17	1.000
25	C9504	phosphorous metabolism	23	22	1.000
26	C9528	repressor	52(53)	77(79)	1.000
27	C9523	activator	58(59)	92(93)	1.000
28	C9490	**anaerobic respiration**	89(91)	161(162)	**0.016**
29	C9372	Transcription related	91(93)	143(146)	0.772

*GCP *values with less than 5% significance probability are indicated in bold type. The ID in the classification scheme by EcoCyc [44] and the corresponding gene function are denoted in the second and third columns, respectively. Two networks in the functions C9448 and C9462 are composed of the same constituent genes with the same connectivity. In the following columns, the numbers of nodes and edges of the analyzed networks are denoted: the original network was constructed based on the information about the relationship between the transcription factor and its regulated genes in EcoCyc, and the analyzed network was constructed from the original network by excluding the genes that were not found in the expression profile data from NCBI GEO (accession number: GSE1107) [25]. The numbers of nodes and edges of the original networks are denoted in parentheses. The graph consistency probability (*GCP*) is denoted in the last column.

The first network (No. 14) is composed of *malT *and its regulated genes. *malT *is involved in maltose transport [28]. Besides the *malT*-regulating network, four networks related to carbon compounds (Nos. 3, 7, 12 and 15) were also included in the examined networks, but they showed no significant probability: the TFs in each network are *araC*, *galR*, *gutM*, and *exuR*, and they regulate products related to the transport of arabinose, galactose, glucitol, and hexuronate, respectively [29-32]. Among the four networks, the *galR *and *exuR*-regulating networks (Nos. 12 and 15) are coordinated in terms of their products: the *exuR *regulatory gene product controls the expression of the galacturonate pathway operons (*exuT*, *uxaC*, *uxuA*, and *uxaB*) [33]. Interestingly, galactose was the least efficiently utilized under anaeorbic conditions, among glucose, lactose, galactose, maltose, maltotriose, and maltohexaose [34]. This fact may be one of the reasons why our method revealed the consistency of network No. 14 with the data, and the lack of consistency of two of the networks, Nos. 12 and 15. In the remaining two networks, Nos. 3 and 7, there are no reasons for their lack of consistency with the present data. Thus, the detection of the network related to maltose metabolism is reasonable, at least in comparison with the galactose- and hexuronate-related networks.

As for the second network (No. 28), the biological function is defined as anaerobic respiration, and its detection is clearly reasonable. The gene encoding the transcription factor in the network is *fnr*, one of the seven global regulators in *E. coli *[35], and the modular controlled by its product, Fnr, encodes proteins involved in cellular adaptation to growth in anoxic environments [36-38]. Since the network is related to adaptations to environmental changes, many genes are comprehensively associated with each other, and the network structure is complex, as seen in Fig 5B. Thus, the consistency of the *fnr*-regulating network with the present data demonstrates the validity of the present method for searching a large-size network consistent with data measured under particular cellular conditions.

It may seem overly strict to estimate the network consistency by the present method. Some other networks besides the two detected networks might be operating under anaerobic conditions. However, the strictness of the consistency estimation is one of the prerequisites for exploring unknown networks. The falsely detected networks should be excluded as much as possible, and the detection of a few definite network candidates may serve as the initial step for investigating the unknown networks that are unexpected, in terms of biological knowledge. In addition, the strictness for consistency estimation is easily modified by setting the selection degree with a significance probability. As a result, the present method reveals the strictly consistent networks with the expression profiles measured under specific conditions, and will be useful to find the activated network candidates among many given networks.

### Merits and Pitfalls of the Present Method

The present method successfully evaluates the consistency of a network with the artificial and actual data, which is expressed as a probability, *GCP*. The *GCP *of each known network is estimated from one set of data in which the constituent molecules of the network were measured under one particular condition. Although a large amount of noise prevents a confident estimation of the *GCP*, the present method is robust in terms of the data sampling dimensions, the parameters in the method, and the network structure variation. The plausibility of the structure variation and scale is illustrated by the detection of actual networks for the simple network of the SOS response and the large and complicated network for anaerobic respiration. Thus, the present method is feasible to evaluate the consistency of the networks with a set of data measured under particular conditions.

The present method may be further applied to various analyses of biological issues. One example is a simple extension of the demonstration shown in the preceding section, as follows. Assume that we know more than two distinctive cell stages, and that we can measure the data of the constituent molecules in different stages. Then, we evaluate the consistency of a set of known networks with the respective data. By this evaluation, we may detect the activated networks, among the known networks that are specific to the respective cell stages. For example, the present method may address the problem of which known networks are activated in progressive diseases and in cell differentiation processes. Thus, the present method will be useful to investigate the network variation in various cell stages responding to different environments. Another example is a utilization of the graphs generated in GEV modeling. Assume that we know a network model for a biological phenomenon, and that a few molecules have been newly detected, and are responsible for the phenomenon. Then, we face the issue of how the newly detected molecules should be connected to the previous network. In this situation, our method may present a solution. A new network is tentatively constructed, by connecting the newly detected molecules into the previous network with the full use of biological knowledge, and then the consistency of the tentative network is estimated with the data measured under the conditions where the relationship of the new molecules with the phenomenon was found. If the *GCP *shows the significance probability, then the network is a promising model for the phenomenon. If not, then we can list some network candidates with the significance probability that commonly share the structure of the previous network, among the generated networks for the GEV distribution. Note that the present method aims to evaluate the consistency between the known network structures and the measured data. Thus, the network inference without any given network structures is beyond the present study. At any rate, these two examples will be demonstrated by appropriate networks and data in the near future.

In terms of the methodology, the present method is a rational extension of the previous study based on linear regression [18], by the combination of the Gaussian network and the extreme value distribution. Indeed, the application range on the network structure is expanded, from simple networks with two-layer relationships to more complex networks with multiple-layer relationships. In addition, the present method is complementary with the d-sep test; the graph consistency is estimated for the associations between variables (existence of edges in the graph) in our method, and in contrast, no associations between variables (no edges in the graph) are considered in the d-sep test [14]. However, the d-sep test failed to select the activated networks: when we set the significance probability to 5%, 27 networks among the 29 networks were consistent with the data measured under specific conditions, and only two networks were not (see additional file 7: d-sep test and SEM for 29 network structures). Interestingly, SEM also failed (see also the additional file 7): 27 networks among the 29 networks were consistent with the data, and one of the two remaining networks could not be evaluated, due to a numerical calculation violation. Thus, our method may be appropriate for tightly estimating the graph consistency in comparison with the d-sep test and SEM. Furthermore, our method differs from the d-sep test in a strict sense. The present method is based on the generation of artificial graphs in the estimation of graph consistency with the measured data, while the d-sep test is based on the direct hypothesis of a population distribution [14,16]. Thus, our method is an asymptotic approach, and is similar to various methods for model selection in network inference, such as various Bayesian network models [8,9]. Note that the present *GCP *is an occurrence probability, and definitely differs from the model selection procedure by using the scores that show a relative difference.

The consistency of a model with the observed data also reminds us of the identifiability problem in the compartmental models for tracer kinetics [39]. The identifiability problem addresses the issue of whether the unknown parameters can be determined uniquely or non-uniquely from the tracer data. Although a systematic algorithm for the identifiability problem was proposed regardless of the model structure [40], its application is limited to the ideal context of noise-free data. Recently, we have partially exploited the identifiability problem algorithm to treat data including noise [41]. Indeed, a network including a cyclic relationship has been examined to estimate the consistency with noisy data. Although this method has a limitation of the network size to smaller than 10 nodes and 15 edges, another method with a symbolic approach may partly compensate for the statistical approach presented here for the limitation of the network structure.

## Conclusion

We have proposed a novel method to estimate the consistency of a given network with the measured data as a probability (*GCP*: graph consistency probability), based on the Gaussian network and the generalized extreme value distribution. The performance of the present method was validated by application to artificial graphs with simulated data and actual graphs with measured data from *Escherichia coli*. The plausible evaluation of the consistency between the network structures and the corresponding measured data promises to help reveal the network structure variations depending on the environments in a living cell, as well as to form a bridge between the static network from the literature and the corresponding measurements.

## Methods

### Data Generation for Simulation

We generates the numerical data according to a standard statistical procedure [16]. The data for 10 nodes with 50 sampling dimensions, {*X*_*kl*_, for *k *= 1,2,...10, and *l *= 1,2,...50}, are generated by using the following structural equations that correspond to the parent-descent relationships in Fig. 1:

(1) $$\{\begin{array}{l} X_{1l}=N(0,\sigma )\\ X_{2l}=N(0,\sigma )\\ X_{3l}=N(0,\sigma )\\ X_{4l}=\alpha_{1,4}X_{1l}+N(0,\sigma )\\ X_{5l}=\alpha_{1,5}X_{1l}+N(0,\sigma )\\ X_{6l}=\alpha_{2,6}X_{2l}+\alpha_{3,6}X_{3l}+N(0,\sigma )\\ X_{7l}=\alpha_{4,7}X_{4l}+N(0,\sigma )\\ X_{8l}=\alpha_{4,8}X_{4l}+N(0,\sigma )\\ X_{9l}=\alpha_{6,9}X_{6l}+\alpha_{7,9}X_{7l}+N(0,\sigma )\\ X_{10l}10=\alpha_{9,10}X_{9l}+N(0,\sigma ) \end{array}$$

where *N*(0, *σ*) means a value that follows a normal distribution with a zero mean and a standard deviation of *σ*, and *α*_*i*,*j *_is a path coefficient relating variables *i *and *j*. Here, we set *σ *to 0.1, and the following parameterization was used: *α*_*i*,*j *_= 0.5. Thus, we obtain a graph and examine the corresponding data to estimate their consistency with the graph. Note that the above data generated by linear equations may not precisely reflect the measured data underlying various non-linear relationships. Here, we adopted the linear relationships as the first approximation to test the performance of the present method. The performance for the complex relationships will be tested by actually measured data.

### Recursive Factorization of Causal Graph

Suppose a causal graph is a directed acyclic graph (DAG), *G*(*V*_*i*_, *E*_*j*_), where *V*_*i *_is a vertex (*i *= 1, 2, ..., *n*_*v*_) and *E*_*j *_is an edge (*j *= 1, 2, ..., *n*_*e*_) in the graph. The DAG can be factorized into subgraphs according to the parent-descent relationships [15]. Then, the joint density function *f*(*X*_*i*_), corresponding to *V*_*i *_for the graph *G*, can be factorized into the conditional density functions according to the graph, as follows:

(2) $$f(X_{1},X_{2},\cdots ,X_{n_{v}})=\prod_{i=1}^{n_{v}}f(X_{i}|pa\{X_{i}\}),$$

where *pa*{*X*_*i*_} is the set of variables corresponding to the parents of *V*_*i *_in the graph.

### Gaussian Network (GN)

The causal graph meets the measured data based on the Gaussian network model [20]. On the assumption that the probability variable *X*_*i *_is subjected to a multiple normal distribution, each conditional function in equation (2) is obtained by linear regression for the measured data of the constituent nodes (molecules) measured at *m *points, i.e.,

(3) $$f(X_{i}|pa\{X_{i}\})=\frac{1}{\sqrt{2\pi \sigma_{i}^{2}}}\exp\left[\frac{-1}{2\sigma_{i}^{2}}\sum_{k=1}^{m}{\left(x_{ik}-\sum_{j=1}^{n_{i}}\beta_{ij}x_{jk}\right)}^{2}\right],$$

where *x*_*ik *_is the measured value of *X*_*i*_, at the *k*-th point, and *n*_*i *_is the number of variables corresponding to the parents of *V*_*i*_. Thus, the joint density function in equation (2) is expressed by the regression for the measured data in equation (3). Finally, the logarithm of the likelihood of the equation (3) is calculated for the measured data as

(4) $$l(G_{0})=\ln\prod_{i=1}^{n_{v}}f(X_{i}|pa\{X_{i}\})=-\frac{1}{2}\sum_{i=1}^{n_{v}}\sum_{j=1}^{n_{i}}\left\{\frac{1}{\sigma_{i}^{2}}\sum_{k=1}^{m}{\left(x_{ik}-\sum_{j=1}^{n_{i}}\beta_{ij}x_{kj}\right)}^{2}+\ln\left(2\pi \sigma_{i}^{2}\right)\right\}.$$

Thus, the GN allows us to quantify a given network into the corresponding numerical value from the measured data, according to the network form. Note that the calculation of likelihood itself requires no assumptions on the relationships between variables. Indeed, the likelihood can be calculated in the case of non-linear regressions, such as spline regression.

### Generalized Extreme Value Distribution (GEV)

Next, we estimate the probability of *l*(*G*_0_) by using the generalized extreme value distribution [21]. First, the log-likelihoods of an ensemble of *n *networks generated according to the *GN *are calculated, and then the maximum log-likelihood is selected from them. The above procedure is iterated *l *times, i.e.,

(5) $$\begin{array}{c} l_{\max}^{1}=Max\left\{l\left(G_{1}^{1}\right),l\left(G_{2}^{1}\right),...,l\left(G_{n}^{1}\right)\right\}\\ l_{\max}^{2}=Max\left\{l\left(G_{1}^{2}\right),l\left(G_{2}^{2}\right),...,l\left(G_{n}^{2}\right)\right\}\\ \cdot \\ \cdot \\ \cdot \\ l_{\max}^{l}=Max\left\{l\left(G_{1}^{l}\right),l\left(G_{2}^{l}\right),...,l\left(G_{n}^{l}\right)\right\} \end{array}$$

The distribution of the maximum values by *l *iterations is expected asymptotically to be a generalized extreme value distribution, i.e.,

(6) $$G\left(l_{\max}\right)=\exp\left\{-{\left[1+\xi \left(\frac{l_{\max}-\mu }{\sigma }\right)\right]}^{-1/\xi }\right\}$$

defined on the set,

$$\left\{l_{\max}:1+\xi \left(\frac{l_{\max}-\mu }{\sigma }\right)>0\right\}$$

where the parameters satisfy -∞ <*μ *< ∞, *σ *> 0, and -∞ <*ξ *< ∞. The model has three parameters: *μ*, *σ*, and *ξ *are a location parameter, a scale parameter, and a shape parameter, respectively. Maximization of the log-likelihood of equation (6) with respect to the parameter vector (*μ*, *σ*, *ξ*) leads to the maximum likelihood estimate for any given dataset, using standard numerical optimization. In the present study, the *R extRemes *package [42] was used to fit the data to the GEV distribution.

Note that the standard likelihood ratio test [43] cannot be applied straightforwardly to a Gaussian network in the present case. This is because the density function of the population and the degrees of freedom in the likelihood ratio test are unclear when maximizing the likelihoods of the generated graphs. In the present method, the GEV distribution of the maximum values of likelihoods in the blocks of generated graphs is adopted analogically, instead of the maximum likelihood in the likelihood ratio test. The utilization of the GEV distribution requires the model fitting to the data, but allows us to set the significance probability arbitrarily, as usual in statistical tests.

Graph Consistency Probability (GCP)

If the goodness of fitness of the maximum values from the generated graphs is ascertained, then the occurrence probability of a given graph (*GCP*: *graph consistency probability*) can be directly estimated by corresponding the *l(G*_0_) in equation (1) to the probability density function of GEV obtained in (6), i.e.

(7) $$P\left(l\left(G_{0}\right)\right)=\int_{\left(G_{0}\right)}^{+\infty }G\left(l_{\max}\right)dl_{\max}.$$

Thus, the present method expresses the consistency in the form of a probability. The probability examines the possibility of whether the tested known networks are activated in the environment where the data were measured. If the probability is small, which corresponds to a large likelihood value, then the data are generated, according to the molecular relationships in the network.

### Actual Networks and Data for High-Throughput Consistency Search

We first classified a transcription factor (TF) and its regulated genes compiled in EcoCyc [44], according to the classification scheme of gene functions . Using the gene sets of the TF and the regulated genes in each function, we next reconstructed the networks: respective networks were reconstructed, so as to form the network structure with as many connections between the genes as possible. Thus, we obtained 130 regulatory networks that are characterized by biological functions. Since some networks were characterized by more than two functions, the 130 regulatory networks were redundant in terms of the connectivity and the constituent genes. Then, 29 networks were kept, after excluding the redundancy and the small networks with less than 8 edges (see Table 1 and additional file 6: 29 network structures analyzed in the present study).

The consistency of each of the 29 networks was estimated with one set of expression profiles measured under 22 different anaerobic conditions (GSE1107) [25] cited from NCBI GEO [45]. The expression profiles were standardized by the average and the standard deviation in each condition, as preprocessing of the measured data. In a few nodes (genes) in the original network constructed from the information in EcoCyc, the corresponding expression profiles were not found in the analyzed data (GSE1107), and the corresponding parts in the network were excluded.

## Authors' contributions

SS carried out the implementation and the calculations, and participated in the design of the study. SA participated in the design of the study, and helped to draft the manuscript. KH conceived of the study, participated in its design and coordination, and drafted the manuscript. All authors read and approved of the final manuscript.

## Supplementary Material

Additional file 1 **Details of the schematic description of the procedure**. The graph factorization at Step 1 and the four GEV-diagnostic plots of the probability plot, the quantile plot, the return-level curve, and the density plot at Step 4 (PDF file) are shown. Click here for file

Additional file 2 **Robustness in terms of data dimensions**. Four GEV-diagnostic plots of the probability plot, the quantile plot, the return-level curve, and the density plot (PDF file) are shown for the 15- and 30-dimension data, respectively. Click here for file

Additional file 3 **Robustness in terms of the parameters**. Four GEV plots (PDF file) are shown when two parameters were set as follows: *l *was set to 25, 50 and 100, and *n *was set to 100, 500, and 1000. Click here for file

Additional file 4 **Robustness in terms of the noise according to the gamma and uniform distributions**. *GCP*(=*P*(*l*(*G*_0_))) for the graph in Fig. 1 was calculated with simulated data according to the gamma and uniform distributions, and the frequencies of *GCP*s are plotted against the probability degree. The horizontal axis indicates the log(*GCP*) value, and the vertical axis is its frequency: black-colored bar, *λ *= 1 in gamma distribution; gray-colored bar, *λ *= 3; striped bar, *λ *= 5; and boxed bar, between 0 and 1 in uniform distribution. Click here for file

Additional file 5 **Robustness regarding the network structure variation**. GEV plots (PDF file) are shown for the three types of network structures in Fig. 3. Click here for file

Additional file 6 **The 29 network structures analyzed in the present study**. The 29 regulatory networks of *Escherichia coli *with more than 8 edges (PDF file) are shown, as constructed from the information on the regulatory relationships between two genes in EcoCyc [44]. Click here for file

Additional file 7 **SEM and d-sep test for 29 network structures**. The 29 regulatory networks of *Escherichia coli *were also tested by SEM and the d-sep test. Click here for file
