# Supplementary figures and images for: Network evaluation from the consistency of the graph structure with the measured data
Source: BMC Syst Biol. 2008 Oct 1;2:84. doi: 10.1186/1752-0509-2-84 (PMC2566979; doi:10.1186/1752-0509-2-84)

Additional file 1 – Details of the schematic description of the procedure

(Step 1)

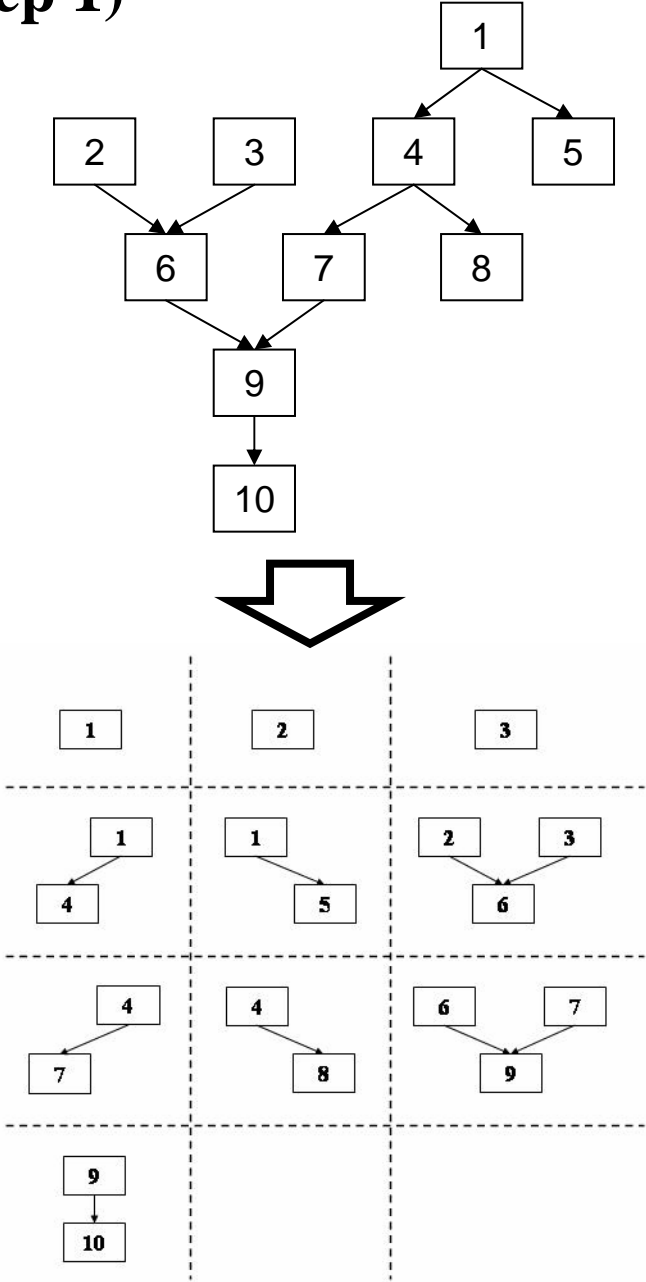

(Step 4)

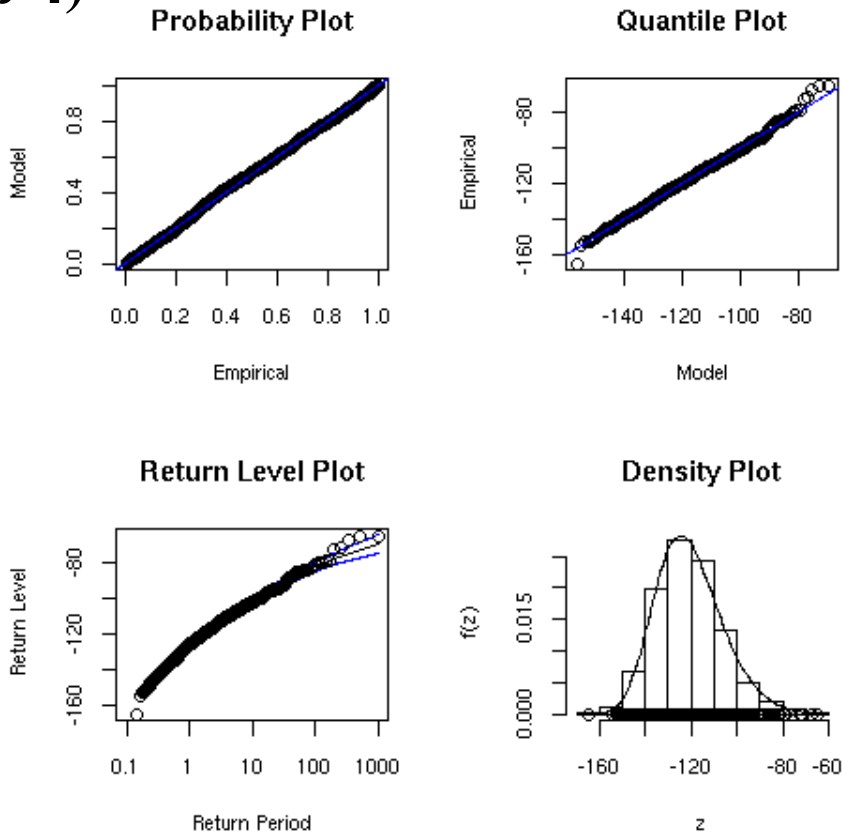

Supplement: Additional file 1 — Details of the schematic description of the procedure. The graph factorization at Step 1 and the four GEV-diagnostic plots of the probability plot, the quantile plot, the return-level curve, and the density plot at Step 4 (PDF file) are shown. [file 1752-0509-2-84-S1.pdf]

# Additional file 2 – Robustness in terms of data dimensions

(15 dimension)

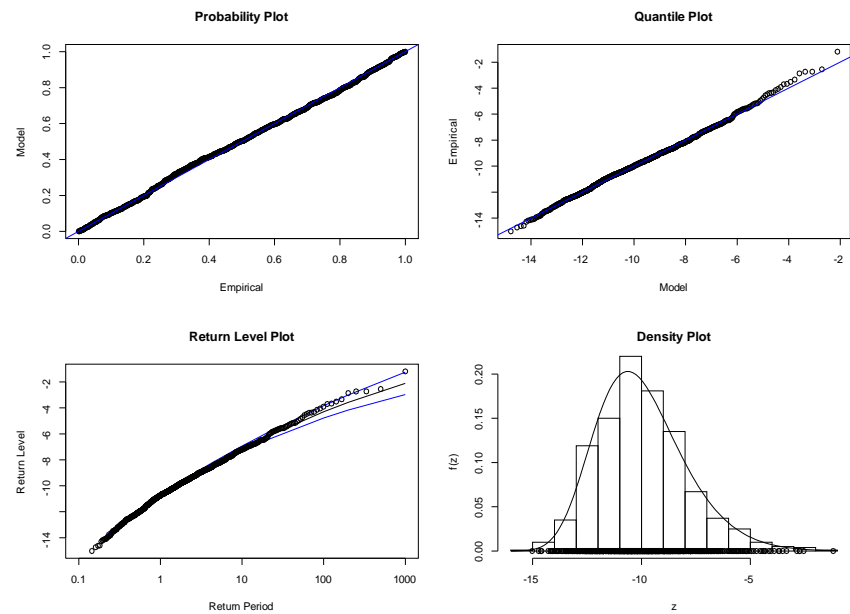

(30 dimension)

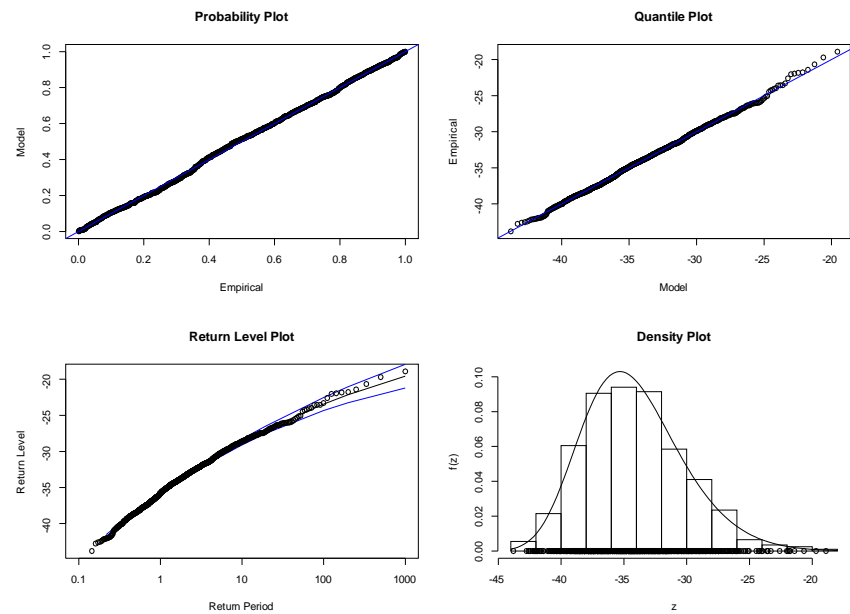

Supplement: Additional file 2 — Robustness in terms of data dimensions. Four GEV-diagnostic plots of the probability plot, the quantile plot, the return-level curve, and the density plot (PDF file) are shown for the 15- and 30-dimension data, respectively. [file 1752-0509-2-84-S2.pdf]

# Additional file 3 – Robustness in terms of the parameters

$n$   
 $l_{max}$

100

500

1000

25

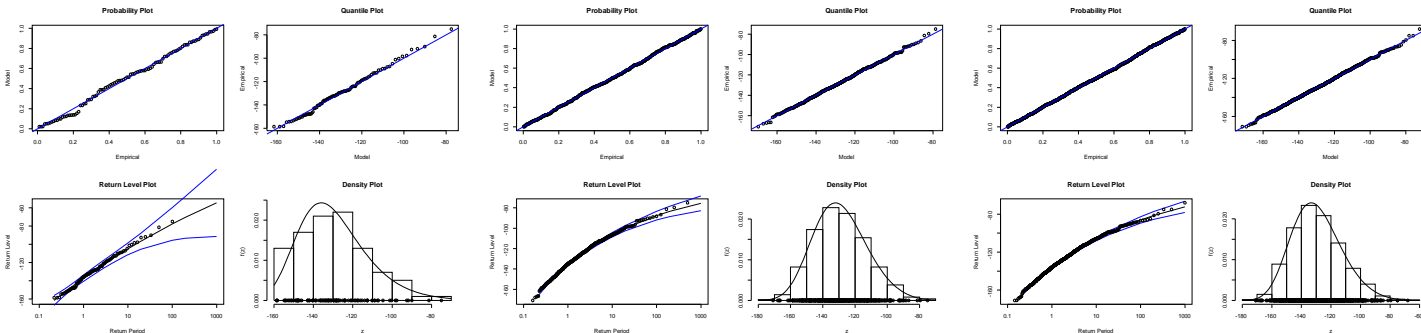

50

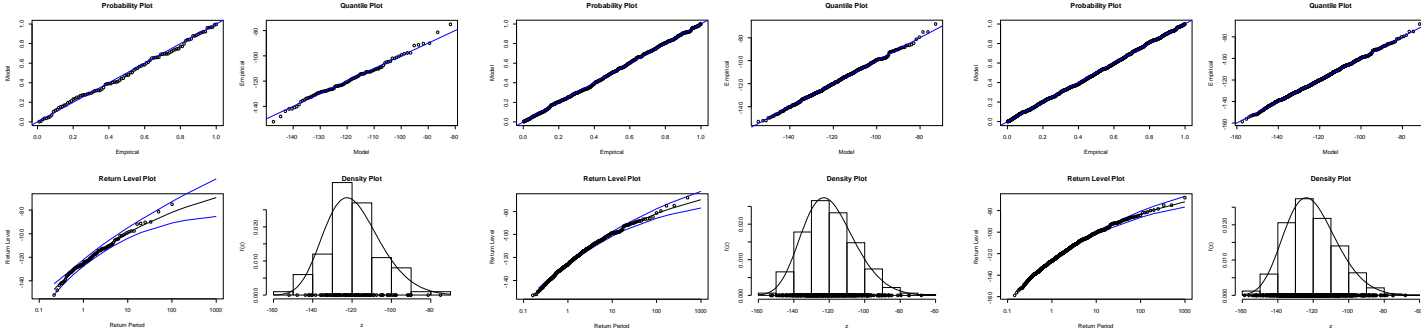

100

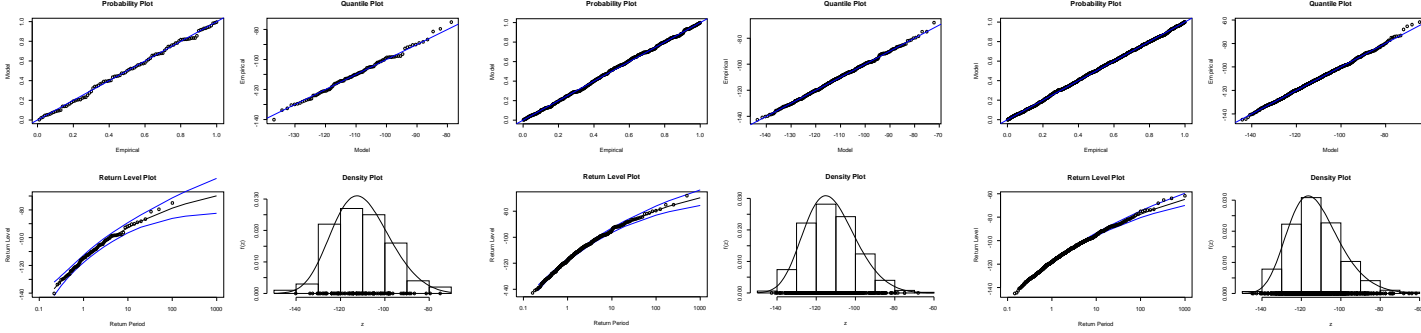

Supplement: Additional file 3 — Robustness in terms of the parameters. Four GEV plots (PDF file) are shown when two parameters were set as follows: l was set to 25, 50 and 100, and n was set to 100, 500, and 1000. [file 1752-0509-2-84-S3.pdf]

# Additional file 5 – Robustness regarding the network structure variation

(A)

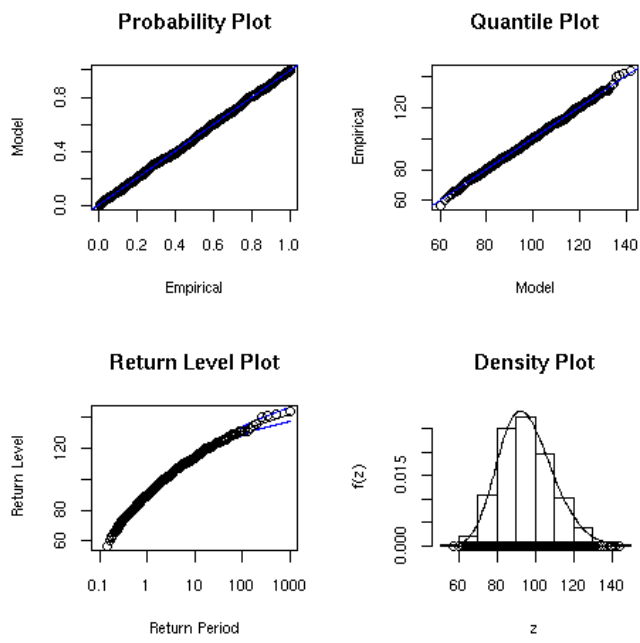

(C)

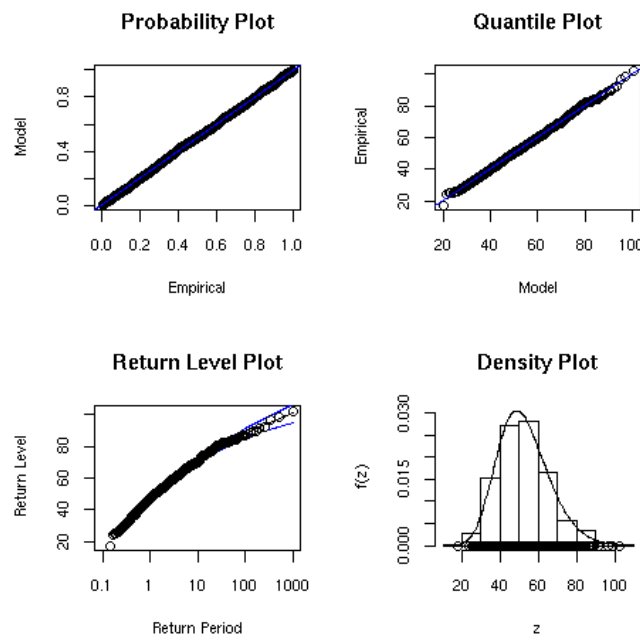

(B)

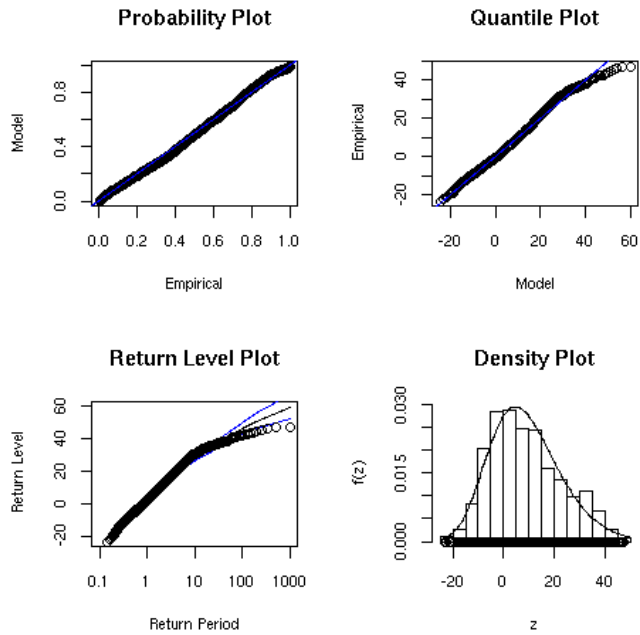

Supplement: Additional file 5 — Robustness regarding the network structure variation. GEV plots (PDF file) are shown for the three types of network structures in Fig. 3. [file 1752-0509-2-84-S5.pdf]
