# Supplementary material for: Network evaluation from the consistency of the graph structure with the measured data
Source: BMC Syst Biol. 2008 Oct 1;2:84. doi: 10.1186/1752-0509-2-84 (PMC2566979; doi:10.1186/1752-0509-2-84)

Additional file 4 – Robustness in terms of the noise according to the gamma and uniform distributions

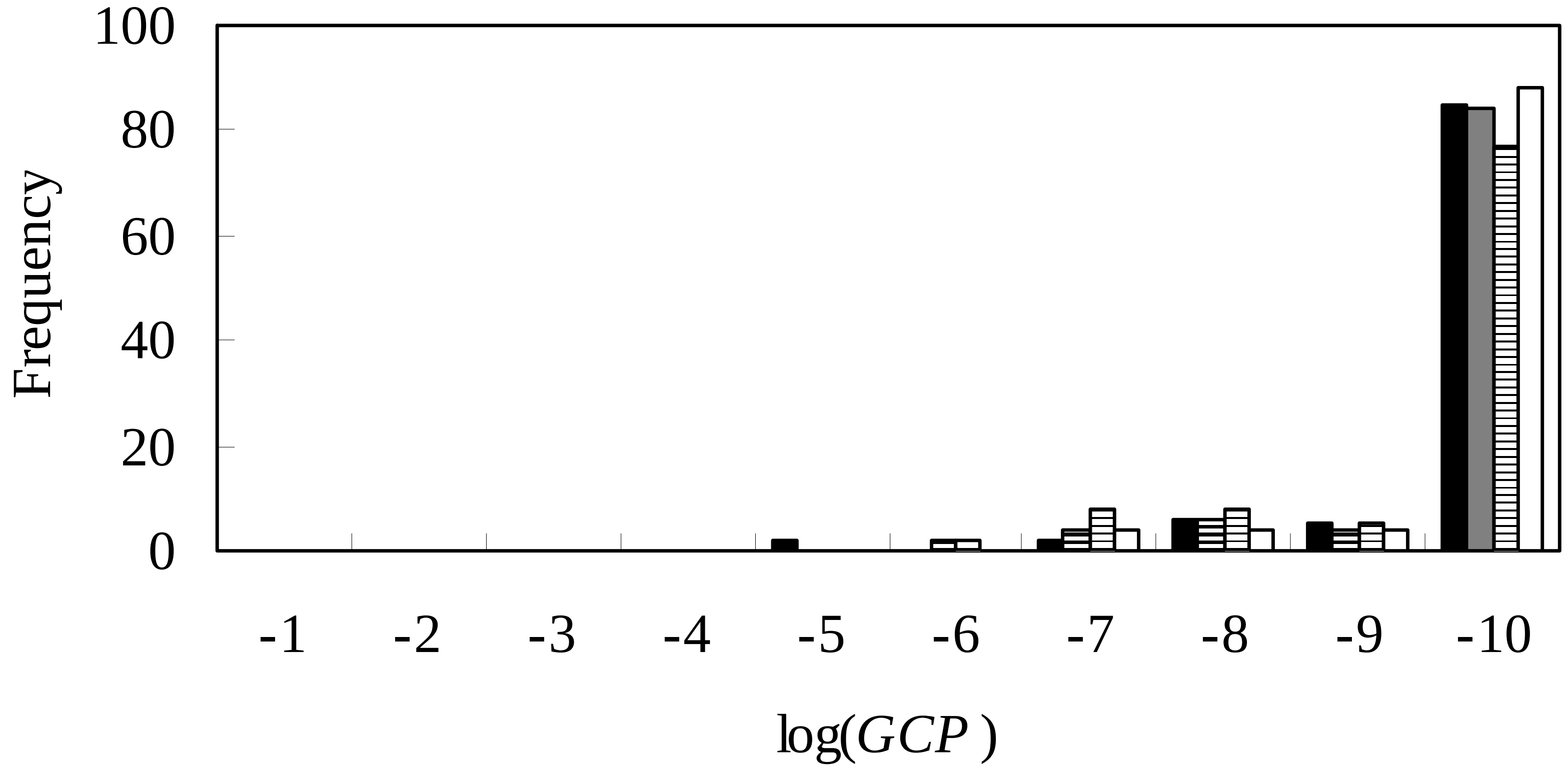

Supplement: Additional file 4 — Robustness in terms of the noise according to the gamma and uniform distributions. GCP(=P(l(G0))) for the graph in Fig. 1 was calculated with simulated data according to the gamma and uniform distributions, and the frequencies of GCPs are plotted against the probability degree. The horizontal axis indicates the log(GCP) value, and the vertical axis is its frequency: black-colored bar, λ = 1 in gamma distribution; gray-colored bar, λ = 3; striped bar, λ = 5; and boxed bar, between 0 and 1 in uniform distribution. [file 1752-0509-2-84-S4.pdf]
