# Supplementary material for: Network evaluation from the consistency of the graph structure with the measured data
Source: BMC Syst Biol. 2008 Oct 1;2:84. doi: 10.1186/1752-0509-2-84 (PMC2566979; doi:10.1186/1752-0509-2-84)
Supplement: Additional file 6 — The 29 network structures analyzed in the present study. The 29 regulatory networks of Escherichia coli with more than 8 edges (PDF file) are shown, as constructed from the information on the regulatory relationships between two genes in EcoCyc [44]. [file 1752-0509-2-84-S6.pdf]

Additional file 6 – The 29 network structures analyzed in the present study

page	ID	description
2	C9333	detoxification
3	C9448	amino acids
4	C9449	carbon compounds
5	C9426	colanic acid (M antigen)
6	C9509	operon
7	C9448, C9462	amino acids, formyl-THF biosynthesis
8	C9449	carbon compounds
9	C9331	motility, chemotaxis, energytaxis (aerotaxis, redoxtaxis etc
10	C9340	flagella
11	C9362	nucleoproteins, basic proteins
12	C9401	tryptophan
13	C9449	carbon compounds
14	C9376	cytoplasm
15	C9449	carbon compounds
16	C9449	carbon compounds
17	C9337	SOS response
18	C9354	DNA repair
19	C9383	arginine
20	C9474	nucleotide and nucleoside conversions
21	C9493	fermentation
22	C9376	cytoplasm
23	C9393	isoleucine/valine
24	C9420	purine biosynthesis
25	C9394	leucine
26	C9504	phosphorous metabolism
27	C9528	repressor
28	C9523	activator
29	C9490	anaerobic respiration
30	C9372	Transcription related

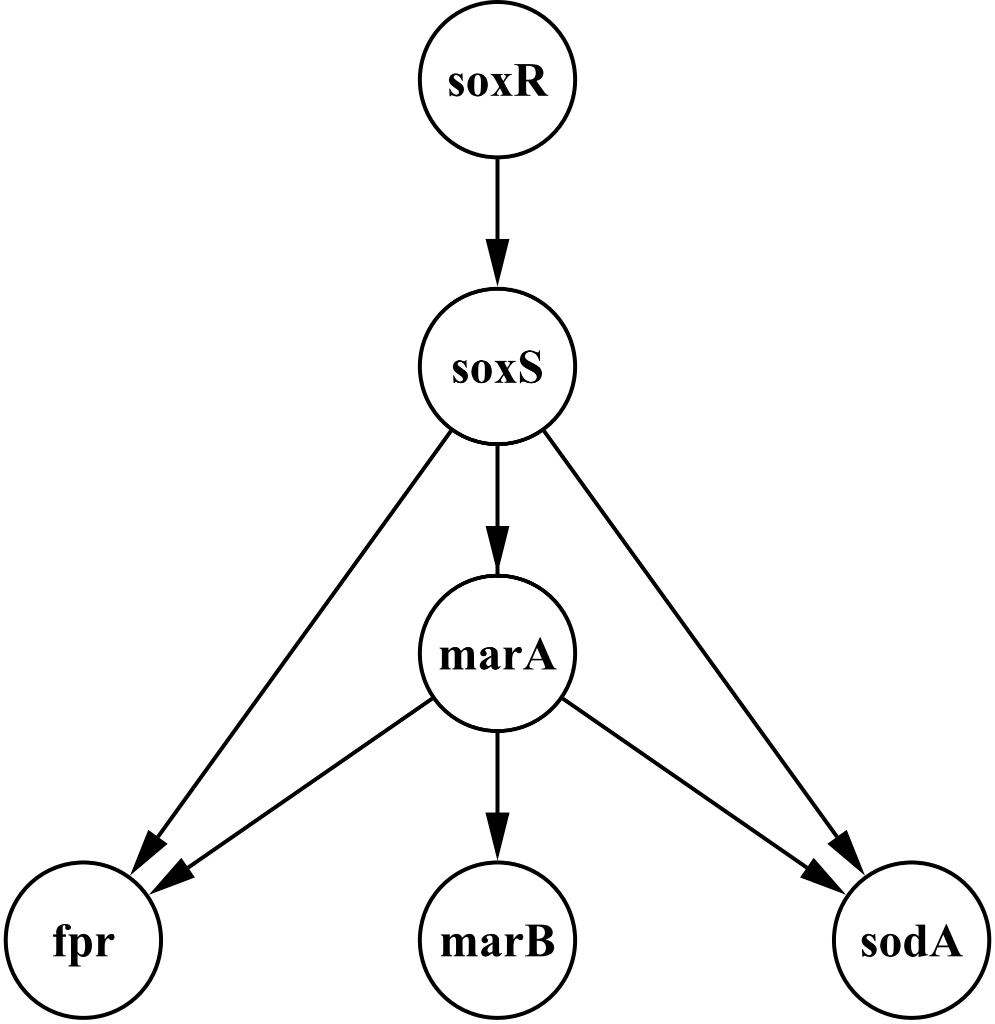
soxR
soxS
marA
fpr
marB
sodA

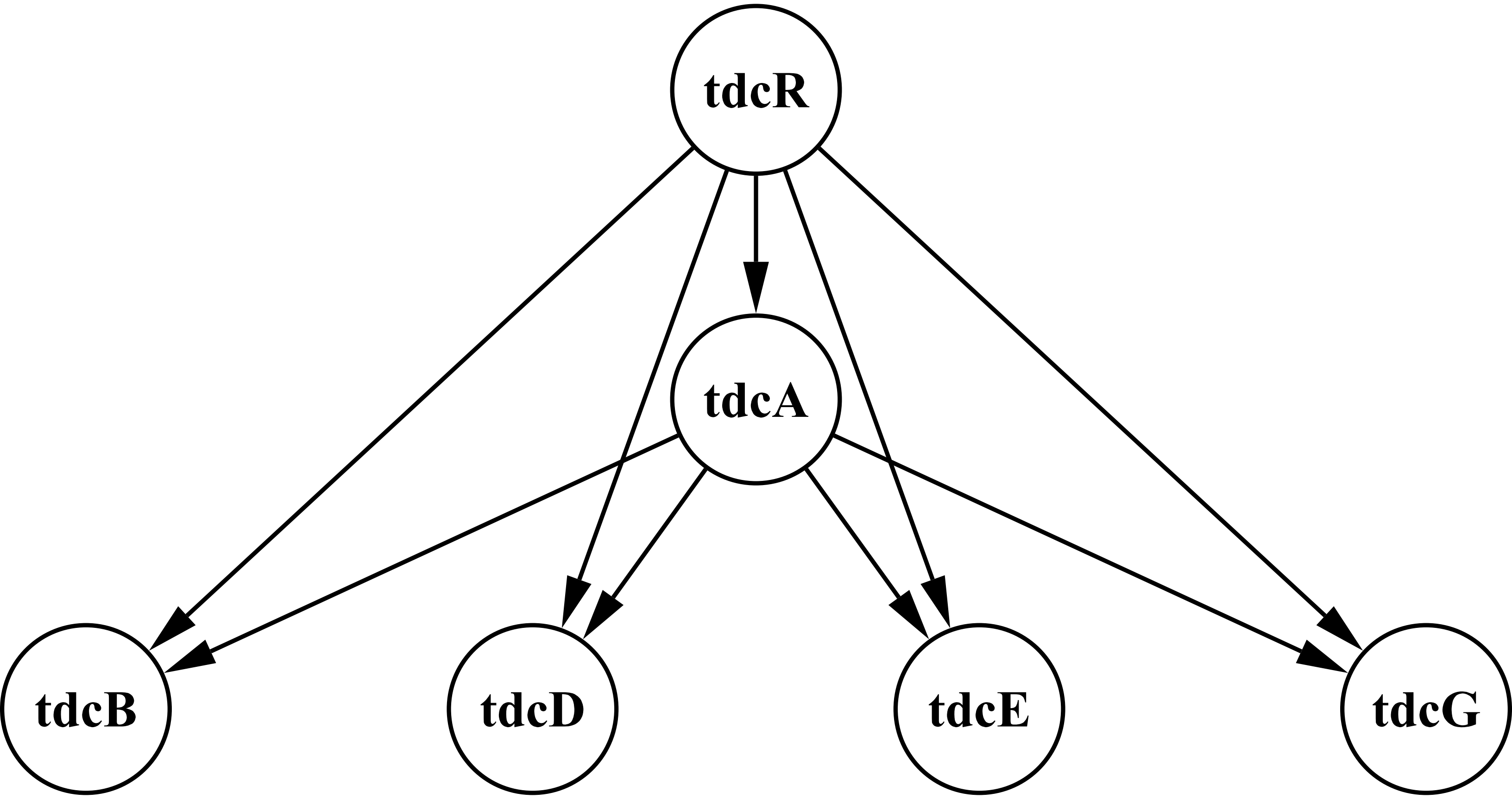
tdcR
tdcA
tdcB
tdcD
tdcE
tdcG

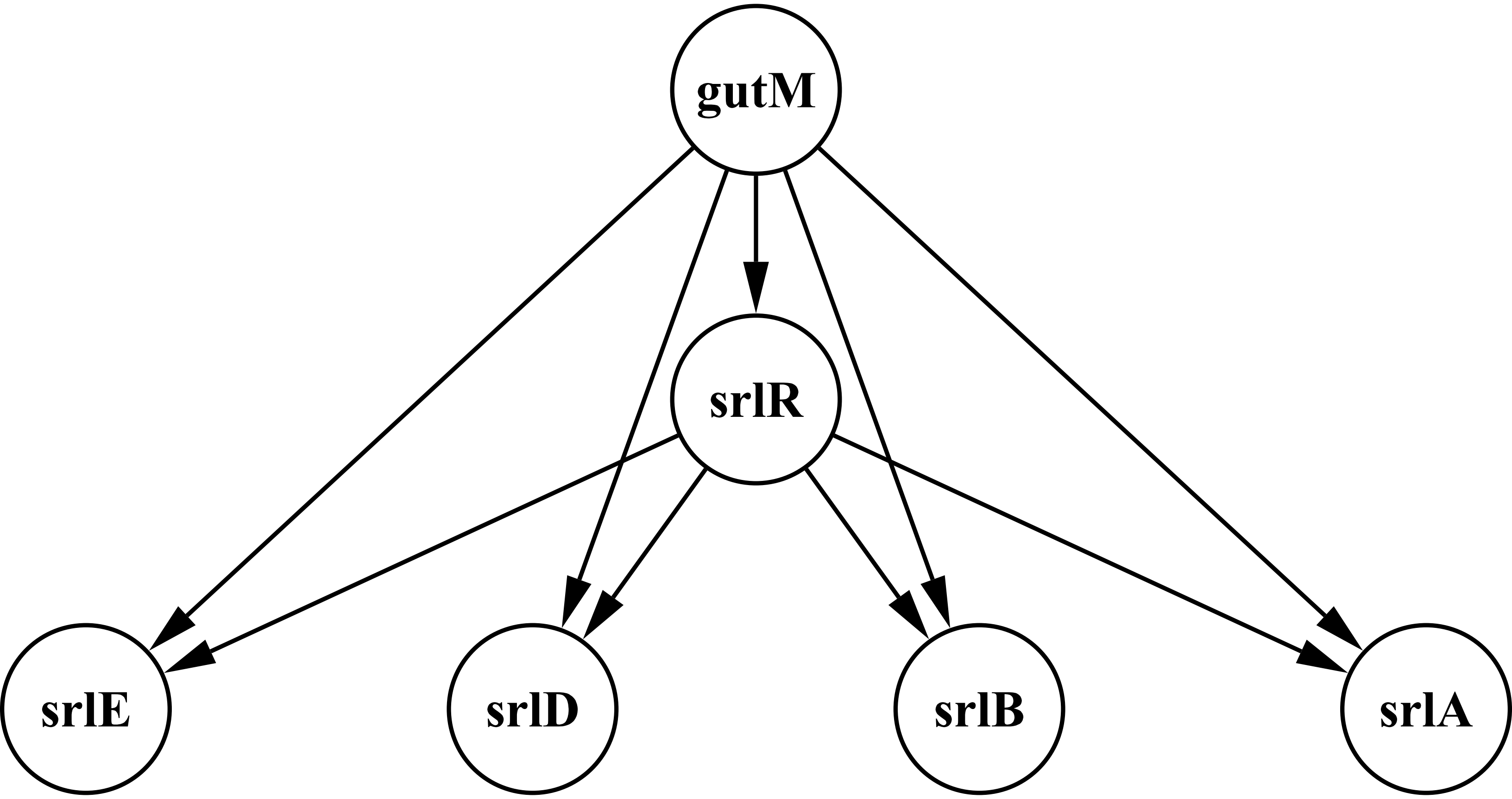
gutM
srlR
srlE
srlD
srlB
srlA

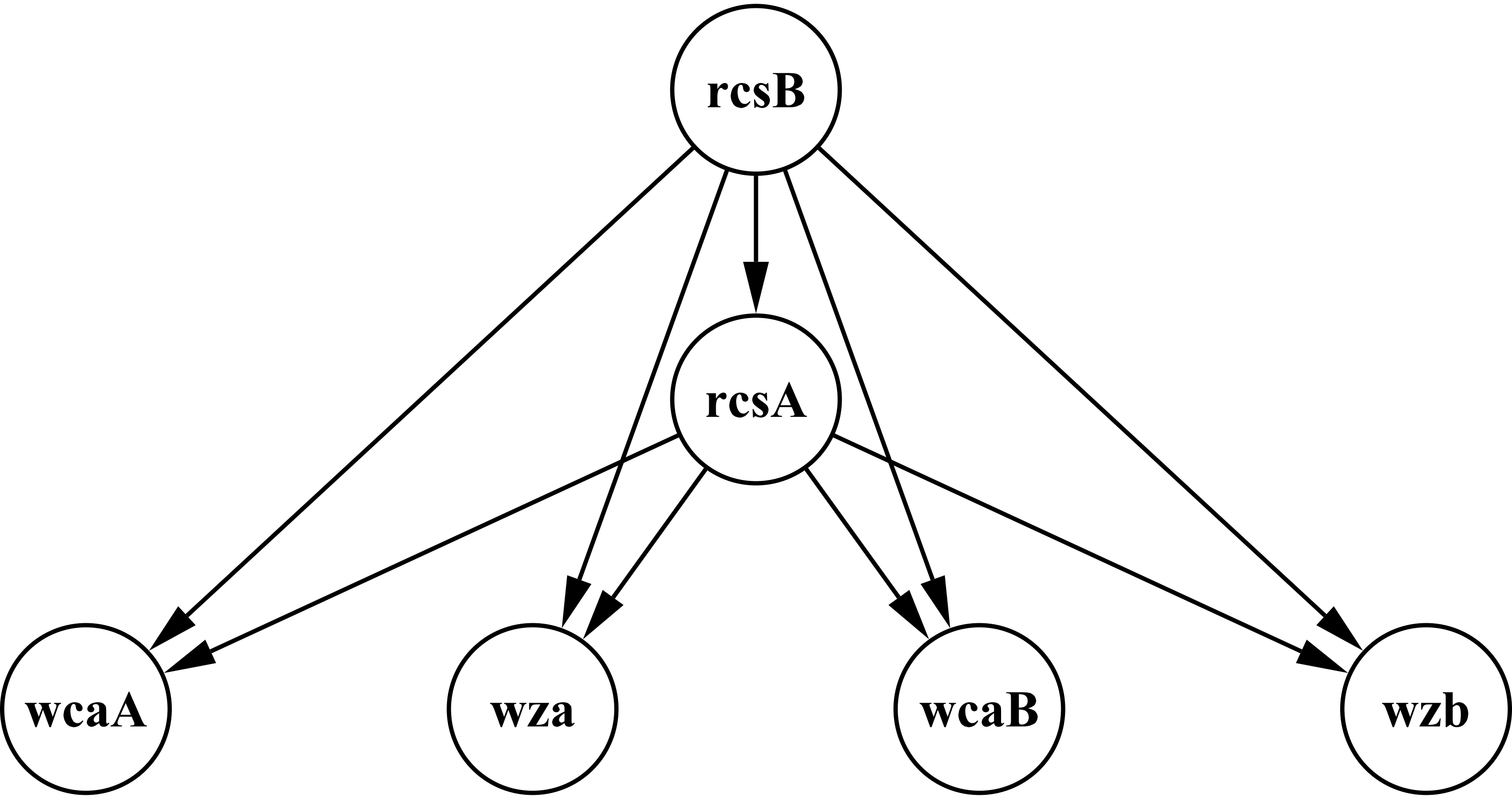
rcsB
rcsA
wcaA
wza
wcaB
wzb

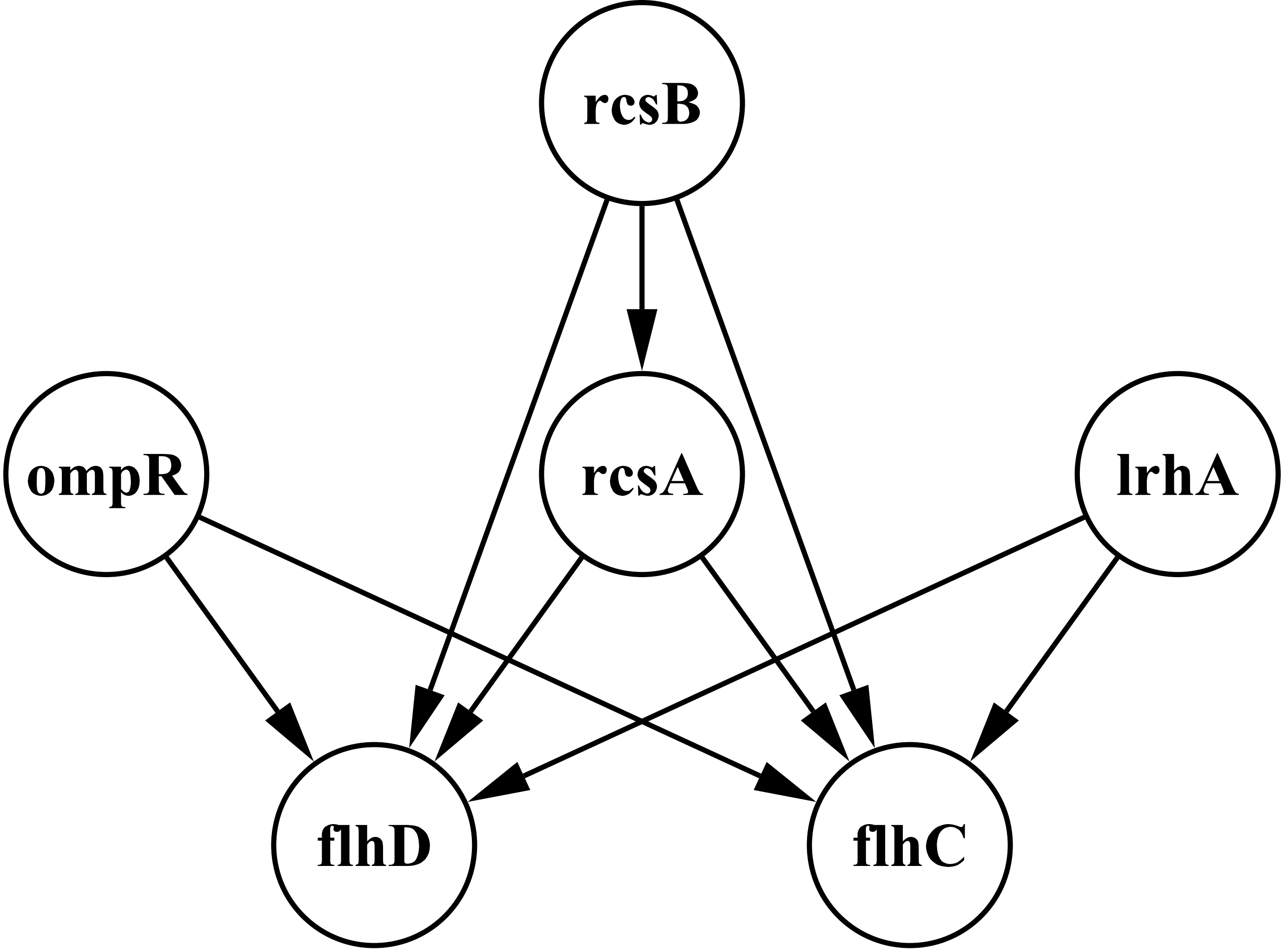
rcsB
ompR
rcsA
lrhA
flhD
flhC

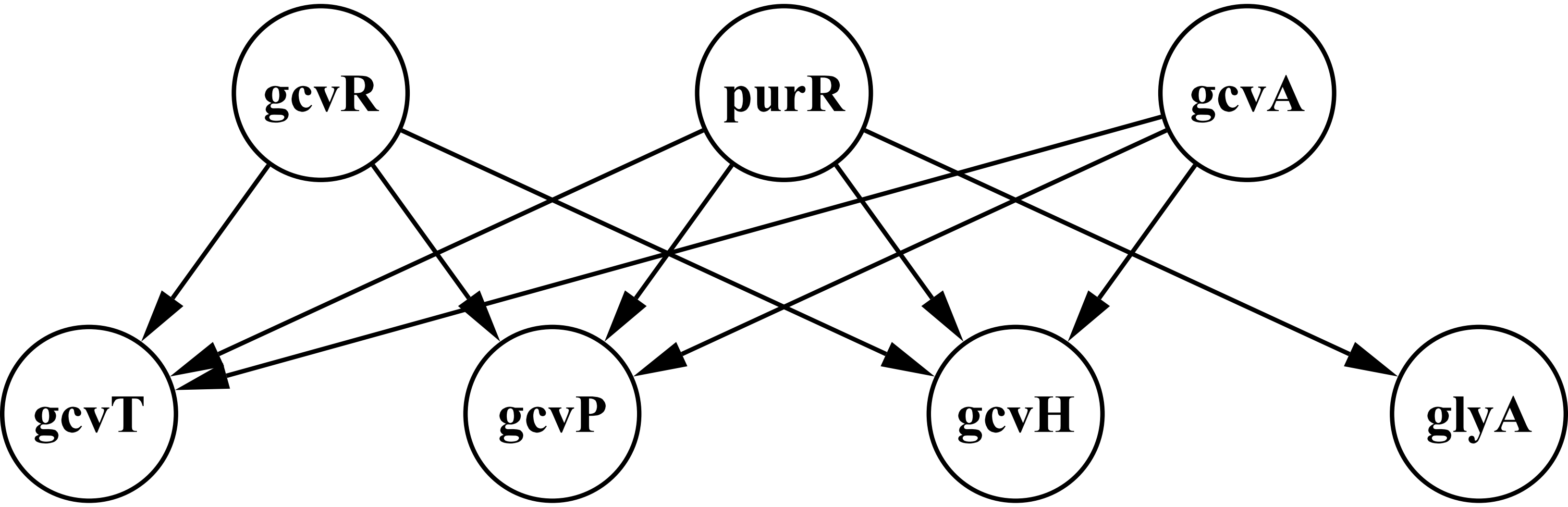
gcvR
purR
gcvA
gcvT
gcvP
gcvH
glyA

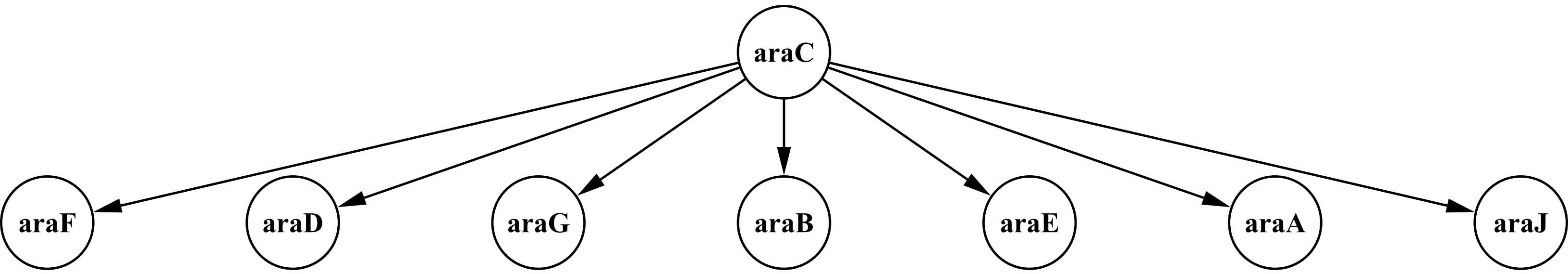
araC
araF
araD
araG
araB
araE
araA
araJ

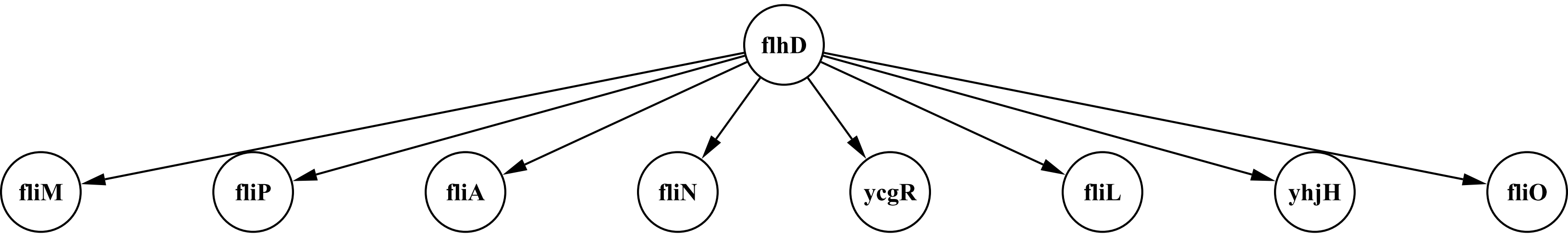
flhD
fliM
fliP
fliA
fliN
ycgR
fliL
yhjH
fliO

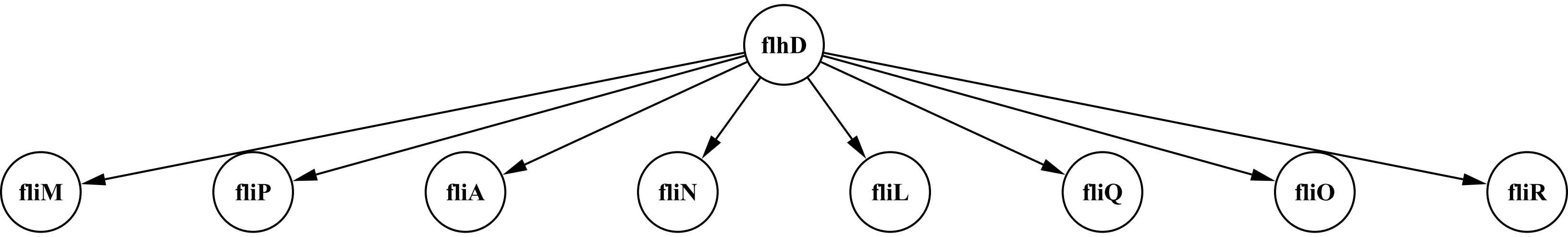

flhD
fliM
fliP
fliA
fliN
fliL
fliQ
fliO
fliR

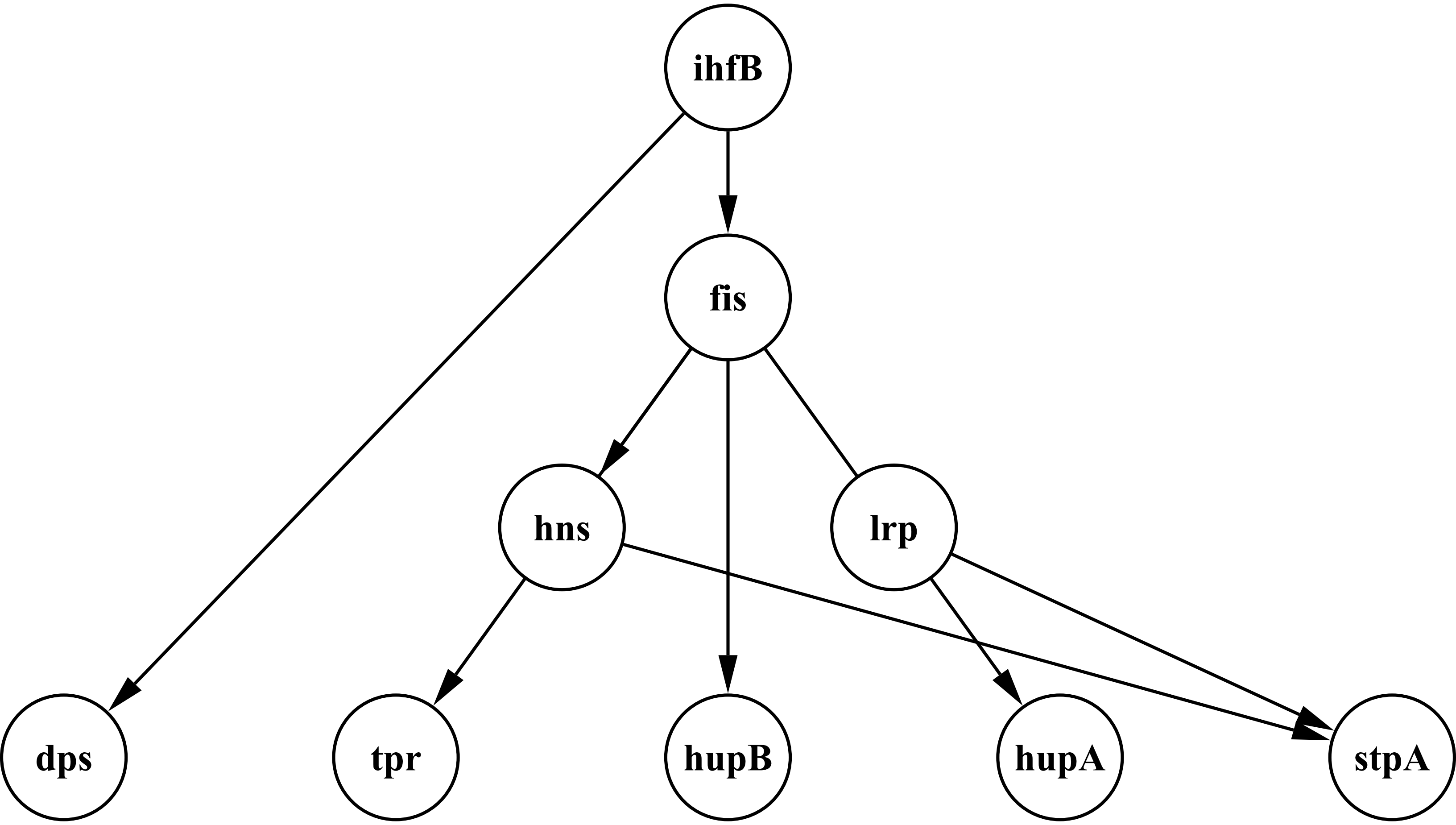
ihfB
fis
hns
lrp
dps
tpr
hupB
hupA
stpA

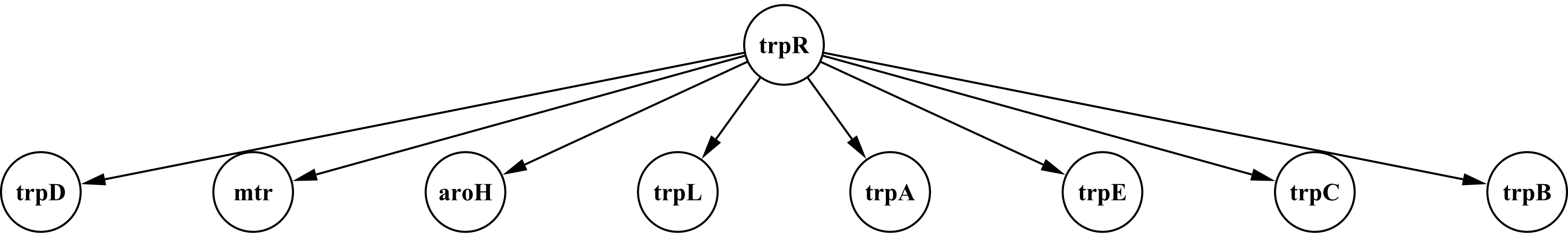
trpR
trpD
mtr
aroH
trpL
trpA
trpE
trpC
trpB

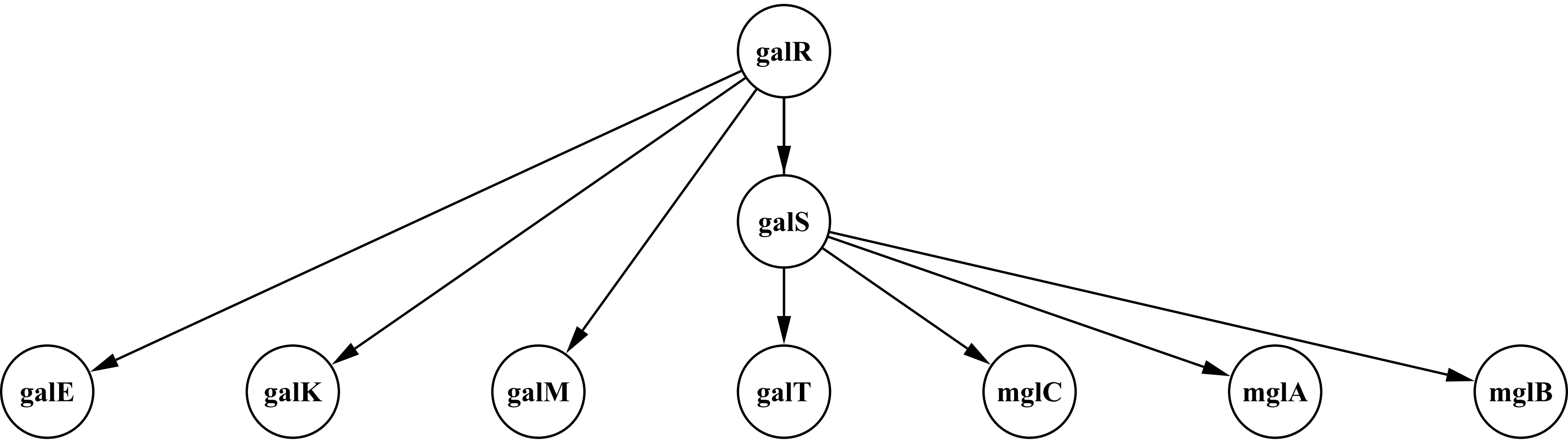
galR
galS
galE
galK
galM
galT
mglC
mglA
mglB

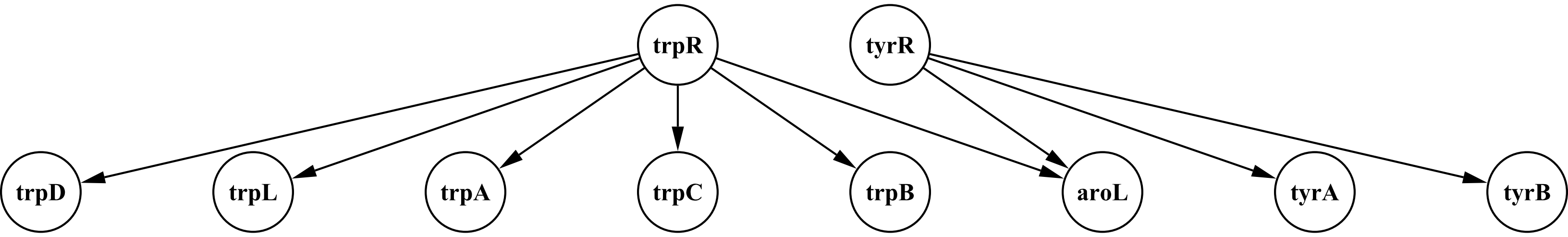
trpR
tyrR
trpD
trpL
trpA
trpC
trpB
aroL
tyrA
tyrB

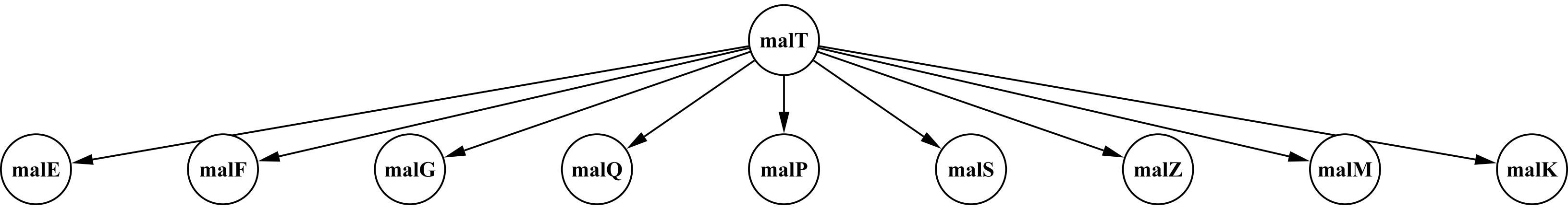

malT
malE
malF
malG
malQ
malP
malS
malZ
malM
malK

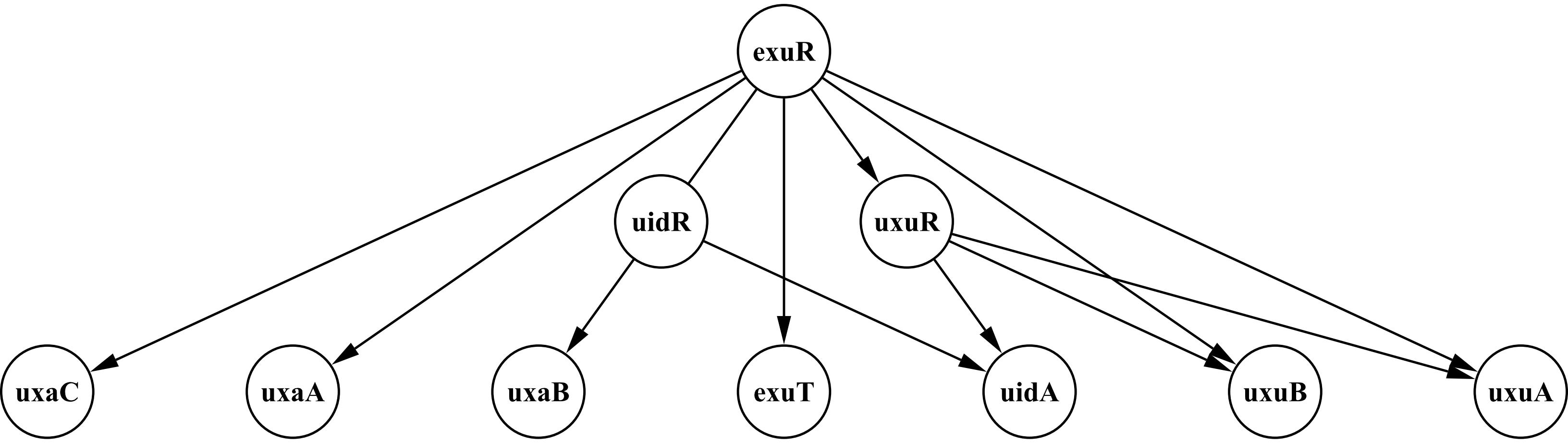
exuR
uidR
uxuR
uxaC
uxaA
uxaB
exuT
uidA
uxuB
uxuA

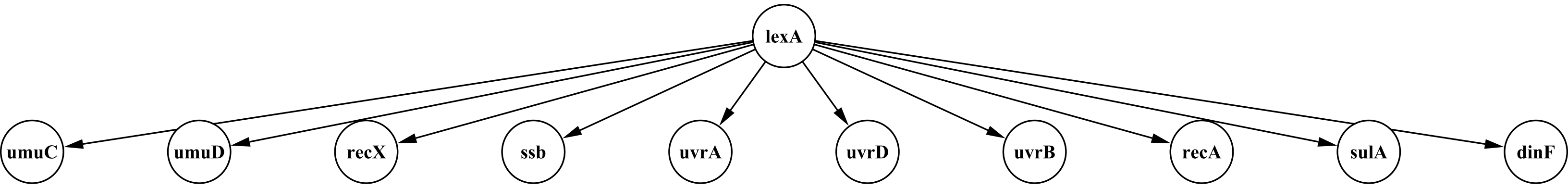
lexA
umuC
umuD
recX
ssb
uvrA
uvrD
uvrB
recA
sulA
dinF

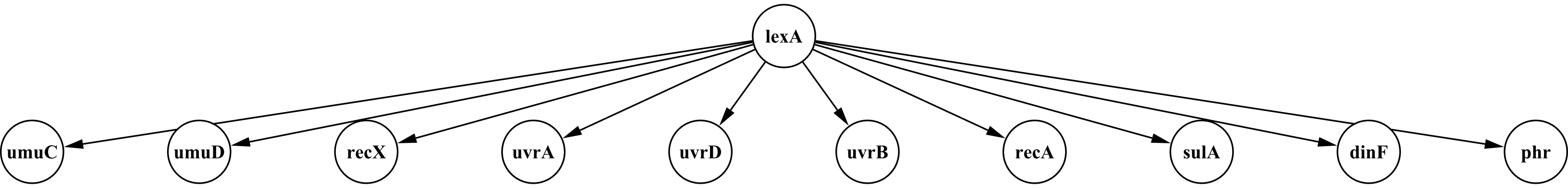
lexA
umuC
umuD
recX
uvrA
uvrD
uvrB
recA
sulA
dinF
phr

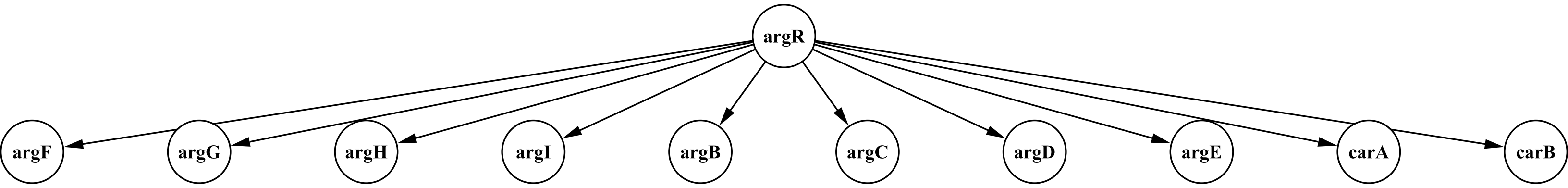

argR
argF
argG
argH
argI
argB
argC
argD
argE
carA
carB

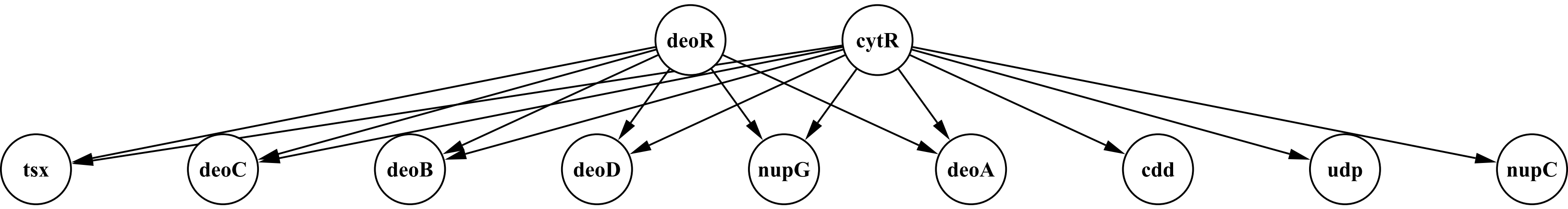
deoR
cytR
tsx
deoC
deoB
deoD
nupG
deoA
cdd
udp
nupC

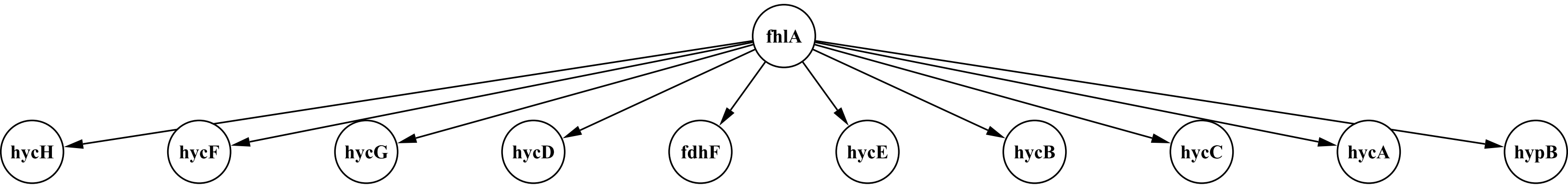
fhlA
hycH
hycF
hycG
hycD
fdhF
hycE
hycB
hycC
hycA
hypB

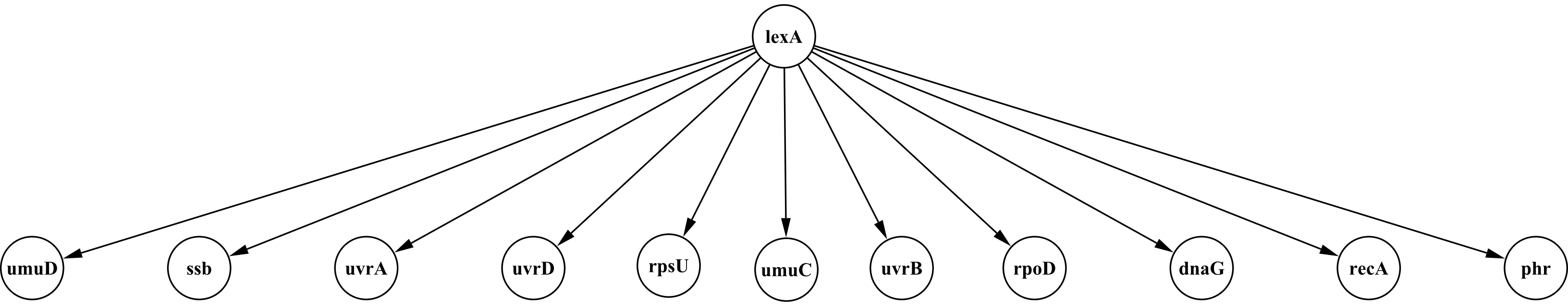
lexA
umuD
ssb
uvrA
uvrD
rpsU
umuC
uvrB
rpoD
dnaG
recA
phr

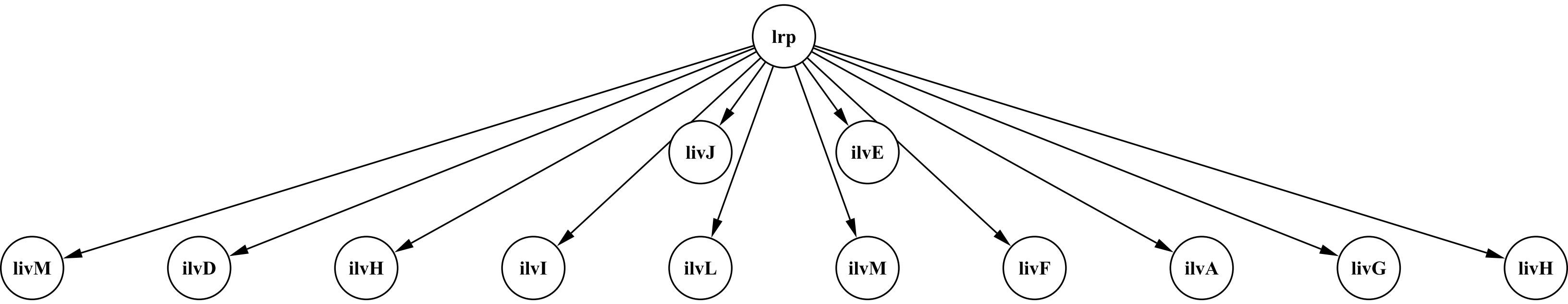

lrp
livJ
ilvE
livM
ilvD
ilvH
ilvI
ilvL
ilvM
livF
ilvA
livG
livH

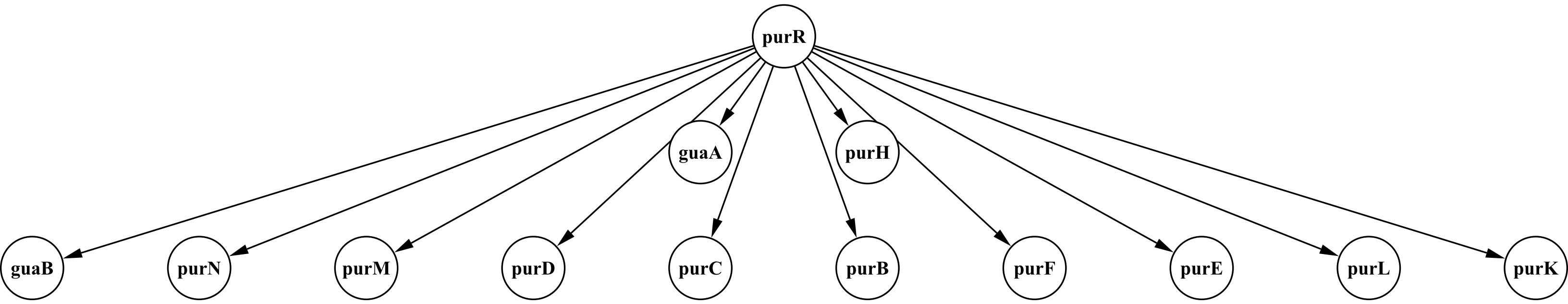

purR
guaA
purH
guaB
purN
purM
purD
purC
purB
purF
purE
purL
purK

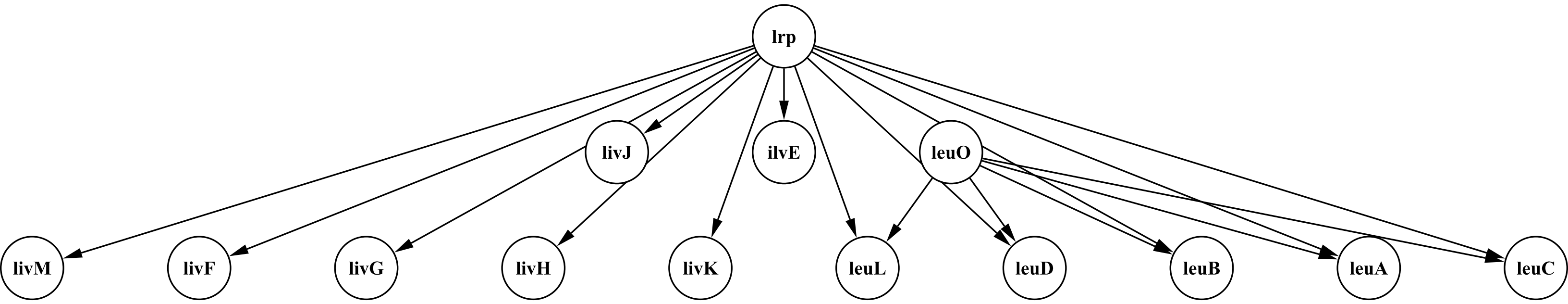
lrp
livJ
ilvE
leuO
livM
livF
livG
livH
livK
leuL
leuD
leuB
leuA
leuC

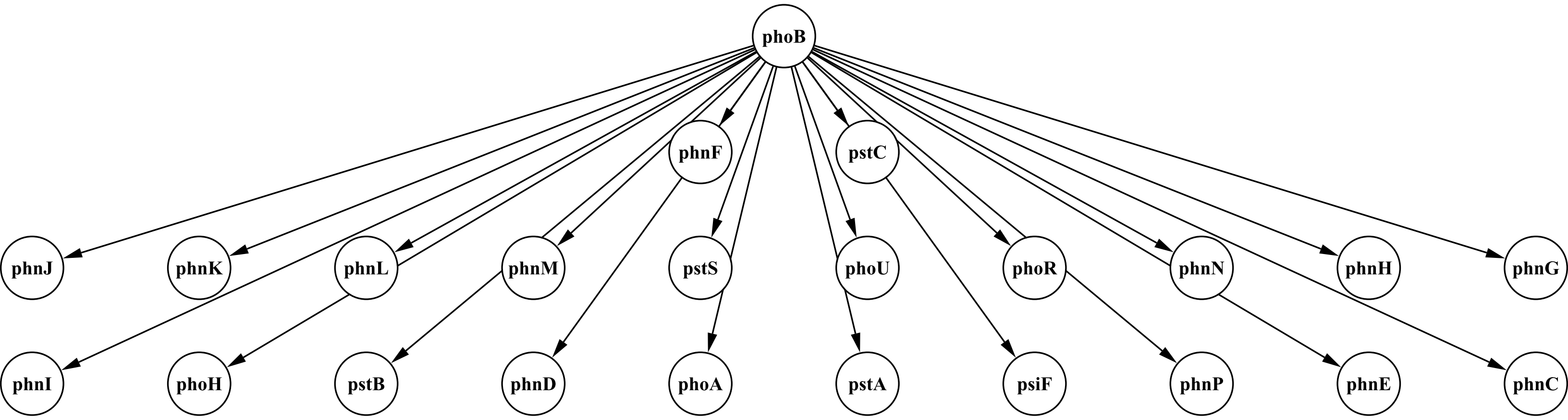
phoB
phnF
pstC
phnJ
phnK
phnL
phnM
pstS
phoU
phoR
phnN
phnH
phnG
phnI
phoH
pstB
phnD
phoA
pstA
psiF
phnP
phnE
phnC

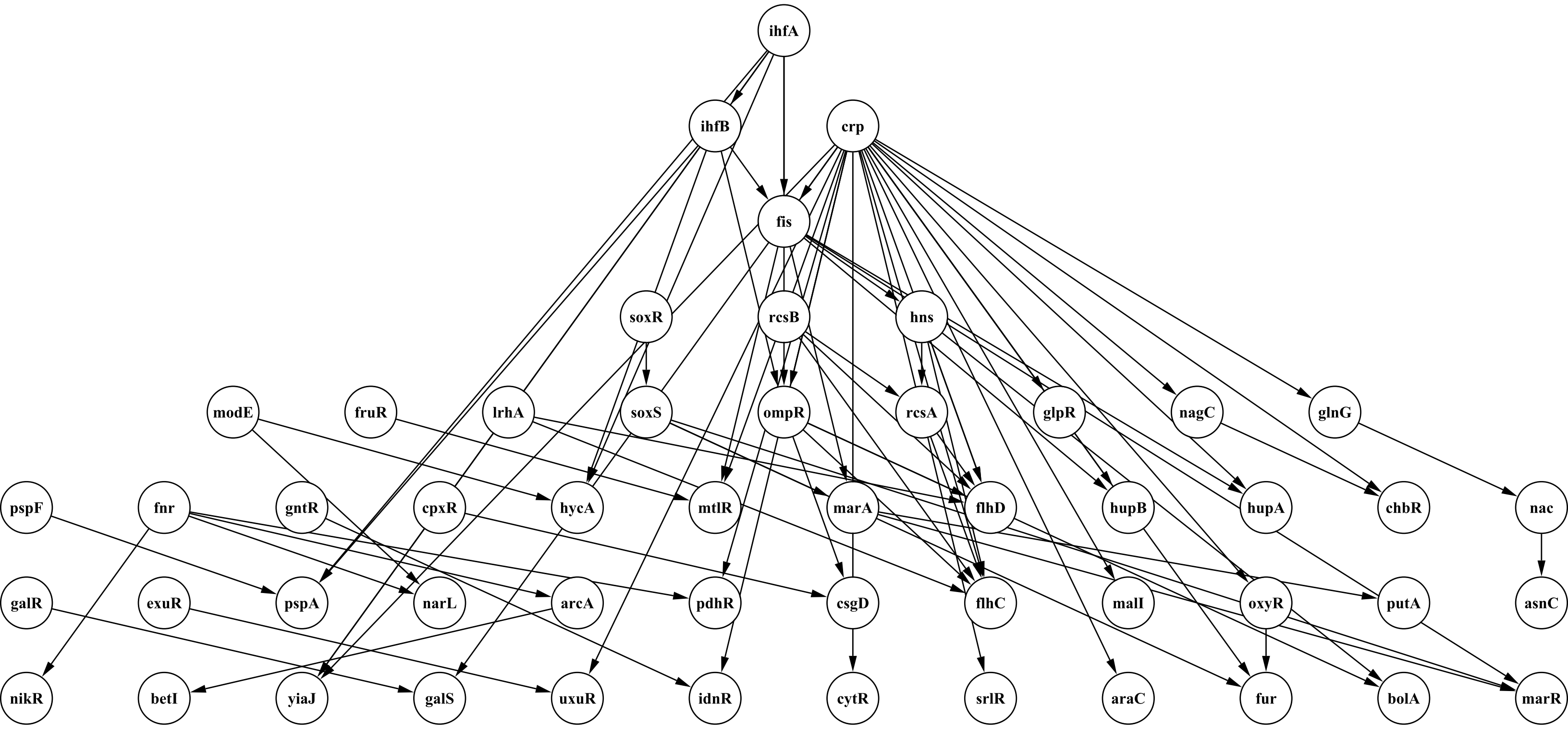
ihfA
ihfB
crp
fis
soxR
rcsB
hns
modE
fruR
lrhA
soxS
ompR
rcsA
glpR
nagC
glnG
pspF
fnr
gntR
cpxR
hycA
mtlR
marA
flhD
hupB
hupA
chbR
nac
galR
exuR
pspA
narL
arcA
pdhR
csgD
flhC
malI
oxyR
putA
asnC
nikR
betI
yiaJ
galS
uxuR
idnR
cytR
srlR
araC
fur
bolA
marR

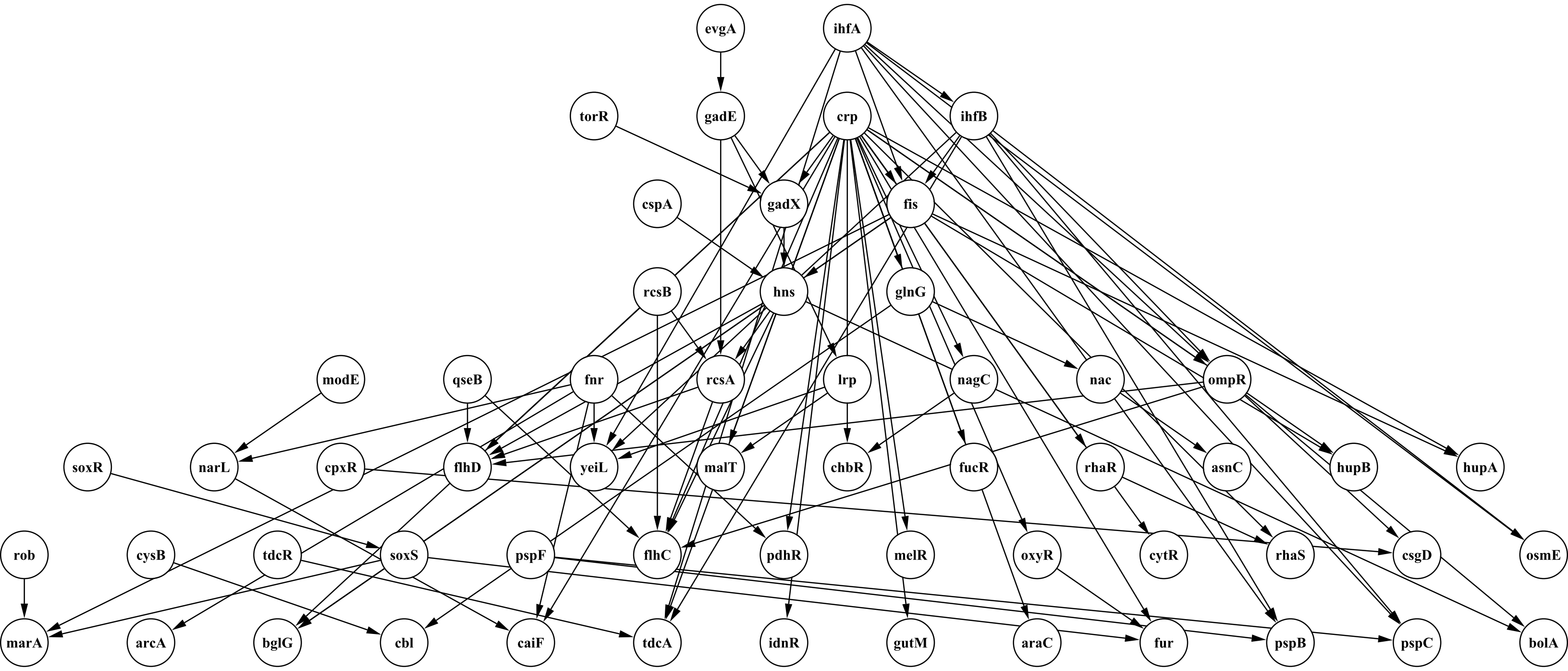
evgA
ihfA
torR
gadE
crp
ihfB
cspA
gadX
fis
rcsB
hns
glnG
modE
qseB
fnr
rcsA
lrp
nagC
nac
ompR
soxR
narL
cpxR
flhD
yeiL
malT
chbR
fucR
rhaR
asnC
hupB
hupA
rob
cysB
tdcR
soxS
pspF
flhC
pdhR
melR
oxyR
cytR
rhaS
csgD
osmE
marA
arcA
bglG
cbl
caiF
tdcA
idnR
gutM
araC
fur
pspB
pspC
bolA

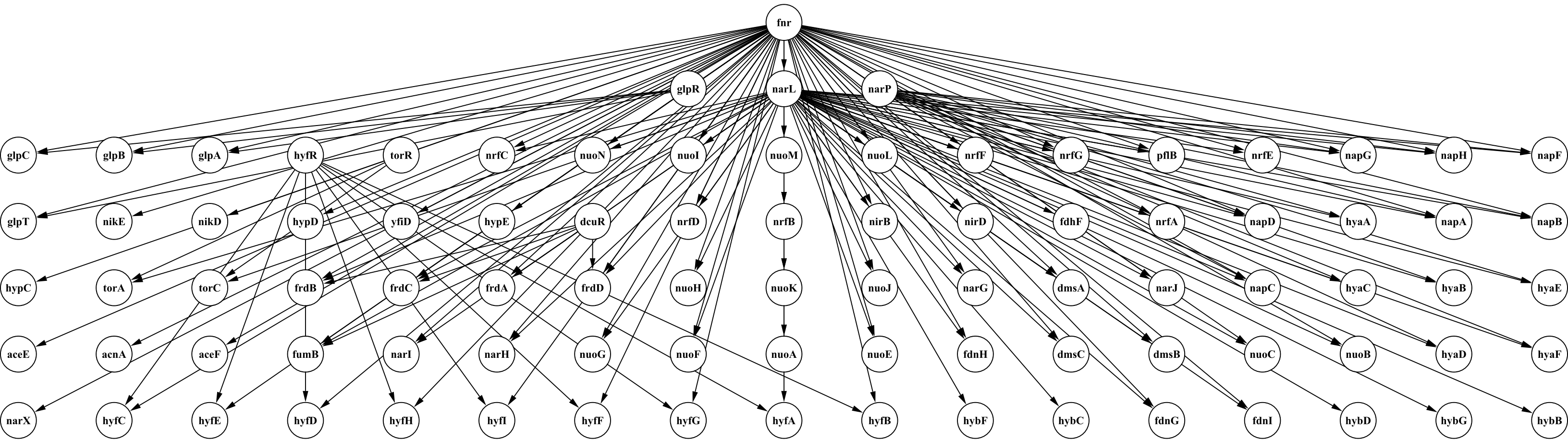
fnr
glpR
narL
narP
glpC
glpB
glpA
hyfR
torR
nrfC
nuoN
nuoI
nuoM
nuoL
nrfF
nrfG
pflB
nrfE
napG
napH
napF
glpT
nikE
nikD
hypD
yfiD
hypE
dcuR
nrfD
nrfB
nirB
nirD
fdhF
nrfA
napD
hyaA
napA
napB
hypC
torA
torC
frdB
frdC
frdA
frdD
nuoH
nuoK
nuoJ
narG
dmsA
narJ
napC
hyaC
hyaB
hyaE
aceE
acnA
aceF
fumB
narI
narH
nuoG
nuoF
nuoA
nuoE
fdnH
dmsC
dmsB
nuoC
nuoB
hyaD
hyaF
narX
hyfC
hyfE
hyfD
hyfH
hyfI
hyfF
hyfG
hyfA
hyfB
hybF
hybC
fdnG
fdnI
hybD
hybG
hybB

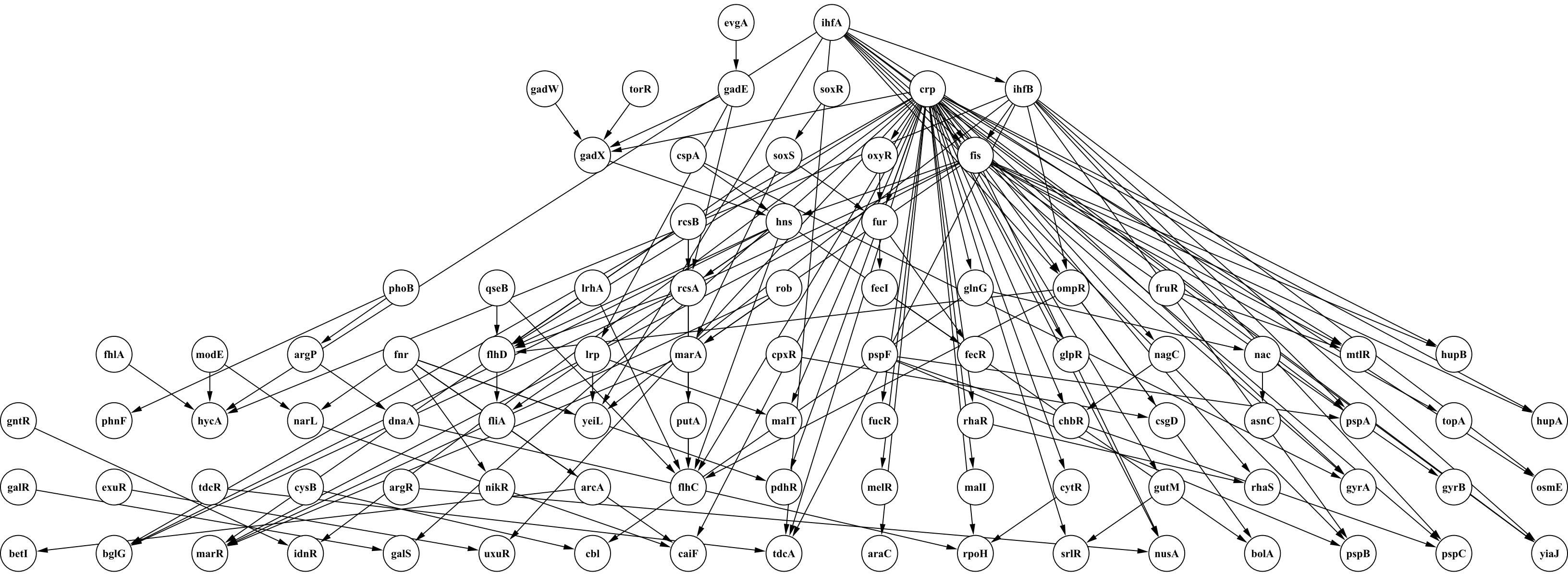
evgA
ihfA
gadW
torR
gadE
soxR
crp
ihfB
gadX
cspA
soxS
oxyR
fis
rcsB
hns
fur
phoB
qseB
lrhA
rcsA
rob
fecI
glnG
ompR
fruR
fhlA
modE
argP
fnr
flhD
lrp
marA
cpxR
pspF
fecR
glpR
nagC
nac
mtlR
hupB
gntR
phnF
hycA
narL
dnaA
fliA
yeiL
putA
malT
fucR
rhaR
chbR
csgD
asnC
pspA
topA
hupA
galR
exuR
tdcR
cysB
argR
nikR
arcA
flhC
pdhR
melR
malI
cytR
gutM
rhaS
gyrA
gyrB
osmE
betI
bglG
marR
idnR
galS
uxuR
cbl
caiF
tdcA
araC
rpoH
srlR
nusA
bolA
pspB
pspC
yiaJ
