# Supplementary material for: Network evaluation from the consistency of the graph structure with the measured data
Source: BMC Syst Biol. 2008 Oct 1;2:84. doi: 10.1186/1752-0509-2-84 (PMC2566979; doi:10.1186/1752-0509-2-84)
Supplement: Additional file 7 — SEM and d-sep test for 29 network structures. The 29 regulatory networks of Escherichia coli were also tested by SEM and the d-sep test. [file 1752-0509-2-84-S7.pdf]

Additional file 7 – SEM and d-sep test for 29 network structures

No.	ID	Description	*GCP*	
			SEM	d-sep test
1	C9333	detoxification	0.193	0.230
2	C9448	amino acids	**0.005**	0.106
3	C9449	carbon compounds	**0.001**	**0.000**
4	C9426	colanic acid (M antigen)	**0.007**	**0.013**
5	C9509	operon	**0.000**	**0.001**
6	C9448, C9462	amino acids, formyl-THF biosynthesis	**0.000**	**0.024**
7	C9449	carbon compounds	**0.000**	**0.001**
8	C9331	motility, chemotaxis, energytaxis	**0.001**	**0.000**
9	C9340	flagella	**0.014**	**0.000**
10	C9362	nucleoproteins, basic proteins	**0.000**	**0.000**
11	C9401	tryptophan	**0.000**	**0.000**
12	C9449	carbon compounds	**0.000**	**0.000**
13	C9376	cytoplasm	**0.000**	**0.000**
14	C9449	**carbon compounds**	**0.000**	**0.000**
15	C9449	carbon compounds	**0.002**	**0.000**
16	C9337	SOS response	**0.000**	**0.000**
17	C9354	DNA repair	**0.000**	**0.000**
18	C9383	arginine	**0.000**	**0.000**
19	C9474	nucleotide and nucleoside conversions	**0.000**	**0.000**
20	C9493	fermentation	**0.000**	**0.000**
21	C9376	cytoplasm	**0.000**	**0.000**
22	C9393	isoleucine/valine	**0.000**	**0.000**
23	C9420	purine biosynthesis	**0.000**	**0.000**
24	C9394	leucine	**0.000**	**0.000**
25	C9504	phosphorous metabolism	NA	**0.000**
26	C9528	repressor	**0.000**	**0.000**
27	C9523	activator	**0.000**	**0.000**
28	C9490	**anaerobic respiration**	**0.000**	**0.000**
29	C9372	Transcription related	**0.000**	**0.000**

The value of '0.000' indicates that the probability was less than 0.0004, and 'NA' indicates that the probability could not be obtained due to the calculation violation.
